# Integration of mechanistic and pharmacokinetic information to derive oral reference dose and margin‐of‐exposure values for hexavalent chromium

**DOI:** 10.1002/jat.3545

**Published:** 2017-10-24

**Authors:** Chad M. Thompson, Christopher R. Kirman, Sean M. Hays, Mina Suh, Seneca E. Harvey, Deborah M. Proctor, Julia E. Rager, Laurie C. Haws, Mark A. Harris

**Affiliations:** ^1^ ToxStrategies, Inc. Katy TX 77494 USA; ^2^ Summit Toxicology, LLP Bozeman MT 59722 USA; ^3^ ToxStrategies, Inc. Mission Viejo CA 92692 USA; ^4^ ToxStrategies, Inc. Austin TX 78731 USA

**Keywords:** benchmark dose (BMD) modeling, hexavalent chromium Cr(VI), margin of exposure (MOE), mode of action, reference dose (RfD), risk assessment

## Abstract

The current US Environmental Protection Agency (EPA) reference dose (RfD) for oral exposure to chromium, 0.003 mg kg^−1^ day^−1^, is based on a no‐observable‐adverse‐effect‐level from a 1958 bioassay of rats exposed to ≤25 ppm hexavalent chromium [Cr(VI)] in drinking water. EPA characterizes the confidence in this RfD as “low.” A more recent cancer bioassay indicates that Cr(VI) in drinking water is carcinogenic to mice at ≥30 ppm. To assess whether the existing RfD is health protective, neoplastic and non‐neoplastic lesions from the 2 year cancer bioassay were modeled in a three‐step process. First, a rodent physiological‐based pharmacokinetic (PBPK) model was used to estimate internal dose metrics relevant to each lesion. Second, benchmark dose modeling was conducted on each lesion using the internal dose metrics. Third, a human PBPK model was used to estimate the daily mg kg^−1^ dose that would produce the same internal dose metric in both normal and susceptible humans. Mechanistic research into the mode of action for Cr(VI)‐induced intestinal tumors in mice supports a threshold mechanism involving intestinal wounding and chronic regenerative hyperplasia. As such, an RfD was developed using incidence data for the precursor lesion diffuse epithelial hyperplasia. This RfD was compared to RfDs for other non‐cancer endpoints; all RfD values ranged 0.003–0.02 mg kg^−1^ day^−1^. The lowest of these values is identical to EPA's existing RfD value. Although the RfD value remains 0.003 mg kg^−1^ day^−1^, the confidence is greatly improved due to the use of a 2‐year bioassay, mechanistic data, PBPK models and benchmark dose modeling.

## Abstract 2

The current US Environmental Protection Agency reference dose (RfD) for chromium (0.003 mg kg^−1^ day^−1^) is based on a 1958 study. A recent 2 year bioassay found that hexavalent chromium [Cr(VI)] in drinking water is carcinogenic to rodents. To assess whether the current RfD is health protective, effects in the recent bioassay were analyzed using physiological‐based pharmacokinetic and benchmark dose modeling. Mechanistic data supported development of RfDs for cancer and non‐cancer effects. Calculated RfDs ranged 0.003–0.02 mg kg^−1^ day^−1^, indicating that the existing RfD is protective of Cr(VI)‐induced non‐cancer and cancer effects.

## 1 INTRODUCTION

Chromium exists in drinking water as trivalent chromium [Cr(III)] and hexavalent chromium [Cr(VI)]. Because of the lack of reducing agents in drinking water and relatively neutral pH, the Cr(VI) species predominates in most supplies. The current US Environmental Protection Agency (EPA) reference dose (RfD) for Cr, 0.003 mg kg^−1^ day^−1^, was last updated in 1998 (US EPA, 1998). The RfD is based on a 1‐year study in F344 rats exposed to ≤25 ppm Cr(VI) in drinking water (Mackenzie, Byerrum, Decker, Hoppert, & Langham, 1958), where 25 ppm was considered the study no‐observable‐adverse‐effect‐level (NOAEL) due to the absence of carcinogenic and non‐carcinogenic effects. More recently, however, exposure to 180 ppm Cr(VI) in drinking water increased oral cavity tumors in F344 rats and exposure to ≥30 ppm Cr(VI) increased the incidence of small intestine (SI) tumors in B6C3F1 mice (National Toxicology Program [NTP], 2008). Environmental monitoring indicates that Cr(VI), which is naturally present in water, is detected at an average concentration of ~0.001 ppm in US drinking water (Ellis, Johnson, & Bullen, 2002; McNeill, Mclean, Parks, & Edwards, 2012; Oze, Bird, & Fendorf, 2007; US EPA, 2017). Notwithstanding the large disparity between environmental and carcinogenic concentrations of Cr(VI), the observation of tumors in the NTP (2008) cancer bioassay necessitates re‐examination of whether the existing RfD value, last updated before the cancer findings, is health protective. To assess this, we use the latest toxicology and mode of action (MOA) data for Cr(VI) to derive RfD values to benchmark against the existing RfD of 0.003 mg kg^−1^ day^−1^.

MOA analysis for cancer outcomes is an important aspect of human health risk assessment (US EPA, 2005). To inform the MOA and risk assessment of oral Cr(VI) exposure, a series of studies were conducted beginning with an overall proposed MOA (Thompson, Haws, Harris, Gatto, & Proctor, 2011), and subsequent 90‐day toxicity studies (Thompson, Proctor, et al., 2011; Thompson et al., 2012), transcriptomic analyses (Kopec et al., 2012; Kopec, Thompson, Kim, Forgacs, & Zacharewski, 2012; Rager et al., 2017), genotoxicity studies (O'Brien et al., 2013; Thompson, Seiter, et al., 2015; Thompson, Wolf, et al., 2015; Thompson, Young, et al., 2015; Thompson et al., 2017), as well as ex vivo gastric reduction studies and pharmacokinetic modeling (De Flora et al., 2016; Kirman et al., 2012; Kirman et al., 2013; Kirman et al., 2016; Proctor et al., 2012). Other genotoxicity studies were conducted in response to early drafts of the NTP 2‐year cancer bioassay (De Flora et al., 2006; De Flora et al., 2008). Based on these studies and others, several scientists and regulatory agencies have concluded that the MOA for the intestinal tumors in mice involves chronic cytotoxicity and regenerative hyperplasia (Becker et al., 2015; Haney, 2015; HealthCanada, 2015; Thompson et al., 2013), and some scientists have proposed RfD values that are protective of both non‐cancerous and cancerous lesions in the mouse SI (Haney, 2015; HealthCanada, 2015; TCEQ, 2016; Thompson et al., 2014).

We previously developed an RfD for Cr(VI) that focused specifically on intestinal effects (Thompson et al., 2014). Since that original publication, additional MOA information has been published that further supports threshold mechanisms for the tumors observed in mice and rats. In addition, new data have been published that better characterize the pharmacokinetics of Cr(VI) reduction in humans, as well as quantitatively account for sensitive populations (Kirman et al., 2016). Herein, we update our risk assessment for the SI and extend our analyses to other endpoints relevant to Cr(VI) exposure. Lesions described in the 2‐year cancer bioassay are reviewed for relevance to setting toxicity criteria and are modeled using physiological‐based pharmacokinetic (PBPK) internal dose estimates and benchmark dose (BMD) modeling methods. Data‐derived extrapolation factors (EFs) are applied to human equivalent doses (HEDs) for derivation of candidate RfD values. The RfD value ultimately selected is designed to be protective against the non‐cancer effects of Cr(VI), as well as cancerous effects in the SI. The oral tumors that occurred in F344 rats primarily at 180 ppm Cr(VI) are analyzed using a margin‐of‐exposure (MOE) analysis. These analyses should be informative for scientists and regulators assessing the health risks associated with oral exposure to Cr(VI).

## 2 METHODS

### 2.1 Data selection

Several risk assessments of Cr(VI) have been conducted in the past few years; however, many of these were completed before the publication of directed research aimed at understanding the pharmacokinetics of Cr(VI) and the MOA for gastrointestinal tumors in rodents (NJDEP, 2009; OEHHA, 2011; US EPA, 2010). The current work therefore focuses on using new MOA and pharmacokinetic data to improve the quantitative risk assessment of Cr(VI). However, a formal systematic review such as those described by the Institute of Medicine (IOM, 2011) or the NTP's Office of Health Assessment and Translation (OHAT, 2015) is beyond the scope of this study. Instead, we expand upon the hazard identification recently conducted by US EPA (2010), which resulted in the quantitative dose–response analysis of the cancer and non‐cancer endpoints listed in Table 1. Reproductive and developmental effects were also considered and are discussed in Section 3.1.2. Only effects observed in chronic exposure studies and reproductive and developmental toxicity studies were considered for RfD derivation.

**Table 1:** Summary of lesions from NTP (2008) considered for dose–response modeling

		Concentration (ppm Cr(VI))				
	Sex	Control	5 (5)^a^	20 (10)	60 (30)	180 (90)
Rats						
Oral tumors	M	0/50	1/50	0/49	0/50	7/49
	F	1/50	1/50	0/50	2/50	11/50
Liver inflammation	F	12/50	21/50	28/50	35/50	39/50
Mice						
Small intestine tumors^b^	M	1/49	3/49	2/49	7/50	20/48
	F	1/49	1/50	4/49	17/49	22/49
Diffuse epithelial hyperplasia	M	0/39	11/43	18/45	42/48	32/40
	F	0/42	16/42	35/48	31/42	42/48
Liver, histiocytic infiltration	F	2/49	15/50	23/50	32/50	45/50
Pancreas, cytoplasmic alteration	M	0/49	1/49	1/50	9/49	8/48
	F	0/48	6/50	6/49	14/50	32/50

F, female; M, male.

a Doses in parentheses are specific to male mice only.

b Combined incidence across intestinal segments of mice surviving 451 days; incidence in each intestinal segment is provided in Table 2.

### 2.2 Dose–response analysis

#### 2.2.1 Physiological‐based pharmacokinetic modeling

**Figure 1:**
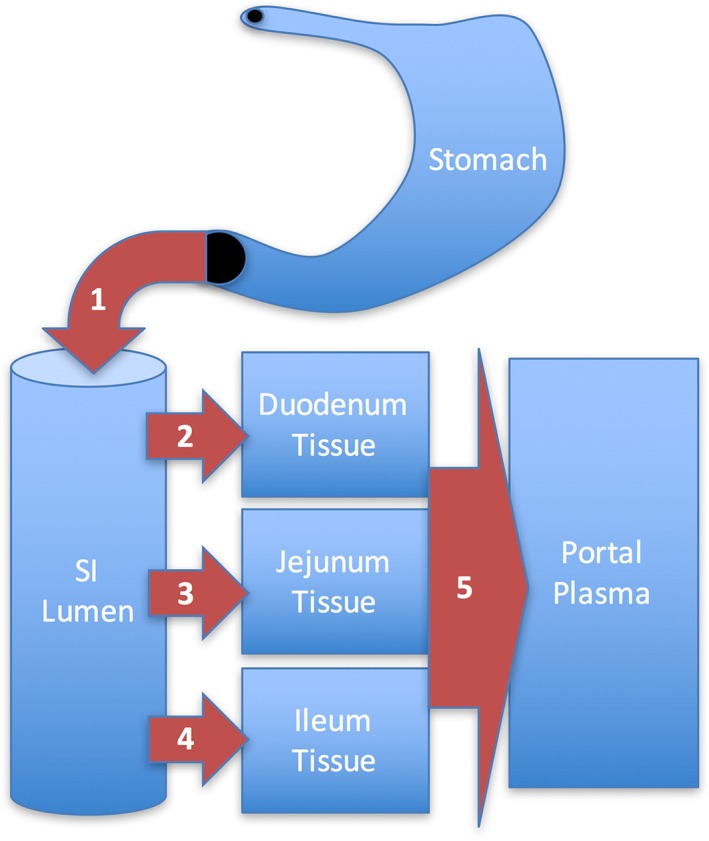
Depiction of flux values estimated with the physiological‐based pharmacokinetic model. (1) pyloric flux (mg Cr(VI) kg^−1^ total SI day^−1^) used for interspecies extrapolation. (2–4) SI segment flux (mg Cr(VI) kg^−1^ SI segment day^−1^) used for dose–response modeling of SI effects. (5) portal flux (mg Cr(V) kg^−1^ bodyweight day^−1^) used for interspecies extrapolation; calculated as the mass of Cr(VI) transferred from all three SI sections, per kg bodyweight. SI, small intestine [Colour figure can be viewed at wileyonlinelibrary.com]

All PBPK modeling was performed in Advanced Continuous Simulation Language Extreme and its add‐in for Microsoft Excel (asclX version 3; Aegis TG). Applied study doses for relevant endpoints were converted to internal dose metrics in target tissues using an updated PBPK model (Kirman, Suh, Proctor, & Hays, 2017). Three internal dose measures were used to support this dose–response assessment (Figure 1): (1) pyloric flux, the amount of Cr(VI) transiting from the stomach lumen to the SI lumen, normalized to SI tissue weight (mg Cr kg^−1^ SI day^−1^); (2) SI sectional flux (the amount of Cr(VI) taken up by enterocytes normalized to SI tissue section weight [mg Cr kg^−1^ SI day^−1^]); and (3) portal flux (the amount of Cr(VI) transiting from gastrointestinal tissue to portal plasma, normalized to bodyweight [mg Cr kg^−1^ bodyweight day^−1^]). Cr(VI) flux values are used to support the assessment, instead of tissue concentrations, because analytical limitations generally preclude measurement of the Cr(VI) species. For this reason, all of the tissue and excretion data used to develop the PBPK model are for total Cr (Cr(III) + Cr(VI)) present in tissues and excreta. Because Cr(VI) is better absorbed than Cr(III), the pharmacokinetics of Cr(VI) are characterized by inference (i.e., increased mass of Cr present for Cr(VI) exposures compared to predictions for Cr(III) exposures). Dose–response relationships for the SI endpoints (hyperplasia, tumors) were assessed using sectional flux of Cr(VI), whereas those for systemic endpoints were assessed using portal flux.

#### 2.2.2 Combining of male and female mice data from the NTP (2008) bioassay

For dose–response modeling of diffuse epithelial hyperplasia and SI tumor formation, male and female mice data were modeled together, because visual examination and statistical analysis (see below) revealed no evidence of sex differences in response to Cr(VI). Recent US EPA guidance states (US EPA, 2012), “Data sets that are statistically and biologically compatible may be combined prior to dose‐response modeling, resulting in increased confidence, both statistical and biological, in the calculated BMD.” Logistic regression was conducted using each response variable as the dependent variable, and dose, sex and the dose × sex interaction as independent variables. The main effect of sex and the dose × sex interaction effect was assessed for relevant sections. Although this was done in our previous assessment (Thompson et al., 2014), changes to the internal dose metric and the change in diffuse epithelial hyperplasia incidence (see Section 2.2.3) warrants re‐examination.

#### 2.2.3 Handling of diffuse epithelial hyperplasia data

The hyperplasia data analyzed herein were taken from tables C4 and D4 in NTP (2008), and are summarized in Table 2. These tables provide the incidence of diffuse epithelial hyperplasia as a function of the number of intestinal segments obtained from necropsied animals. We confirmed these numbers by reviewing the individual animal pathology tables from the study. For most groups, a small number of intestinal sections were not analyzed due to autolysis, or were missing altogether. For example, NTP table C4 and individual pathology tables indicate that only 39 duodena were analyzed from the 50 necropsied male mice in the control group; thus, the incidence of diffuse epithelial hyperplasia should be based on 39 animals (as is the case in NTP table C4). For reasons unclear to the present authors, summary tables in the NTP (2008) report and peer‐reviewed published version (Stout, Herbert, Kissling, et al., 2009) list the incidences of diffuse epithelial hyperplasia based on the number of animals necropsied rather than tissues examined. A second peer‐reviewed publication related to the NTP (2008) cancer bioassay simply lists hyperplasia incidence based on “*N* = 50,” without any specification of whether *N* refers to the number of animals or number of tissues examined (Witt et al., 2013).

**Table 2:** Dose–response data set for mouse intestinal effects using internal dose metrics

Sex	Segment	SI sectional flux (mg kg^−1^ SI day^−1^)	N^a^ (DEH)	DEH	N^b^ (tumors)	Adenomas	Carcinomas	Combined
F	I	1.4E‐05	42	0	49	0	0	0
M	I	2.3E‐05	40	0	49	0	0	0
F	J	1.7E‐04	41	0	49	0	1	1
M	J	2.7E‐04	41	0	49	0	0	0
F	D	3.0E‐03	42	0	49	0	0	0
M	D	3.8E‐03	39	0	49	1	0	1
F	I	2.8E‐02	43	0	50	0	0	0
M	I	3.9E‐02	42	0	49	1	0	1
M	I	1.2E‐01	44	0	49	0	1	1
F	I	2.8E‐01	47	0	49	0	0	0
F	J	2.9E‐01	42	2	50	1	0	1
M	J	4.1E‐01	42	0	49	0	2	2
M	I	5.4E‐01	45	1	50	0	0	0
F	I	8.1E‐01	44	0	49	0	0	0
M	J	1.1E + 00	42	0	49	0	0	0
M	I	1.1E + 00	38	0	48	0	0	0
F	I	1.4E + 00	47	0	49	0	0	0
F	J	2.1E + 00	48	1	49	0	2	2
F	D	3.0E + 00	42	16	50	0	0	0
M	J	3.7E + 00	46	2	50	0	1	1
M	D	3.9E + 00	43	11	49	0	0	0
F	J	5.4E + 00	44	0	49	2	2	4
M	J	7.0E + 00	38	1	48	3	2	5
M	D	7.6E + 00	45	18	49	1	0	1
F	J	9.0E + 00	48	8	49	5	1	6
F	D	1.1E + 01	48	35	49	2	0	2
M	D	1.7E + 01	48	42	50	5	2	6
F	D	2.3E + 01	42	31	49	13	1	14
M	D	2.7E + 01	40	32	48	15	3	16
F	D	3.5E + 01	48	42	49	12	6	17

D, duodenum; DEH, diffuse epithelial hyperplasia; F, female; I, ileum; J, jejunum; M, male; N, sample size.

a Based on incidence from number of animals examined microscopically (tables C4 and D4 of NTP, 2008).

b Based on incidence from number of animals surviving ≥451 days (tables C1 and D1 of NTP, 2008 and tables 5–5 and 5–6 of US EPA, 2010).

Flux values in control animals/segments are based on consumption of Cr(VI) measured in control water samples (0.0053 ppm).

Hyperplasia data from the jejunum were omitted for dose–response modeling, for reasons described previously (Thompson et al., 2014). In brief, hyperplasia incidence in the NTP (2008) bioassay was assessed microscopically via a single 5 μm biopsy taken at the approximate midpoint of each intestinal segment. The mouse duodenum and ileum are each ~9 cm long, whereas the jejunum is ~19 cm long—implying that the biopsy taken in the mid‐jejunum may underestimate hyperplasia in the proximal (duodenal) end of the jejunum, where the chromium level was likely higher than at the midpoint and distal (ileal) end of the jejunum based on chromium levels measured in each segment (Kirman et al., 2012; Thompson, Proctor, et al., 2011). Thus, the relationship between hyperplasia and dose is less certain in the jejunum.

#### 2.2.4 Benchmark dose modeling

Dose–response modeling for adverse effects was conducted using the US EPA's Benchmark Dose Software v.2.6, using the suite of dichotomous models, as well as the dichotomous Hill model. Benchmark response (BMR) values of 5% or 10% extra risk were used to obtain BMD values, along with their corresponding 95% lower confidence limit (BMDL). The slopes were restricted to ≥1, which is done to prevent the estimated dose–response curve from taking on a biologically implausible very steep slope as the dose approaches 0. Per US EPA recommendations (US EPA, 2012), model fits were judged using criteria such as *P* values, scaled residuals, Akaike information criterion, parsimony and visual inspection.

#### 2.2.5 Derivation of human equivalent doses

BMDL values based on internal doses were converted to HEDs using a published human PBPK model for the disposition of ingested chromium (Kirman et al., 2017). For all endpoints, pyloric flux of Cr(VI) was used to support interspecies extrapolations (i.e., determination of HEDs), because pyloric flux can be estimated in humans with much higher confidence than is possible for the other internal dose measures, as discussed in Thompson et al. (2014). The PBPK model was used to support the following extrapolations to facilitate HED predictions for the point of departure (POD) values expressed in terms of SI sectional flux or portal flux (dose measures in brackets reflect those used to support interspecies extrapolation):

- Mouse SI sectional flux → mouse SI total flux → [mouse pyloric flux = human pyloric flux] → HED
- Mouse/rat portal flux → [mouse/rat pyloric flux = human pyloric flux] → HED

The term “mouse SI total flux” represents the sum of the three sectional flux values (duodenum + jejunum + ileum), so that total risk to the entire SI is estimated. For example, for SI endpoints characterized in terms of mouse SI sectional flux, we use the PBPK model to determine the mouse pyloric flux value that occurs when the mouse SI total flux (sum of duodenum, jejunum and ileum fluxes) is equal to the POD value. In this way, a POD corresponding to a 10% response in the SI tissue as a whole will be distributed between the sections based upon the gradient of subtissue doses (e.g., 9% response in duodenum, 0.9% response in jejunum and 0.1% response in the ileum as a hypothetical distribution). This approach assumes that, for a given value of pyloric flux, the dose of Cr(VI) delivered to the SIs and to systemic tissues, as well as their associated risks to the tissue, as a whole, are equivalent for all species.

Pyloric flux in humans was used to estimate human equivalent lifetime average daily doses that correspond to the mouse internal POD values by considering variation in toxicokinetic processes for Cr(VI) as a function of age using the following five age groups: (1) neonate (0–3 months); (2) infant/child (0.25–6 years); (3) youth (6–18 years); (4) adult (18–60 years); and (5) elderly (60–75 years), as described by Thompson et al. (2014). Details on the application of the human PBPK model for the chromium risk assessment are summarized in Appendix A.

### 2.3 Toxicity value derivations

RfD values were derived as follows. The rodent POD was first adjusted by an interspecies EF composed of the toxicodynamic factor (EF_AD_). Per US EPA, PBPK models obviate the need for toxicokinetic factor (EF_AK_), because the HED is computed directly via the PBPK model. The human PBPK model was then used to derive a human equivalent lifetime average daily dose that corresponds to the adjusted rodent internal POD. This POD_HED_ was subsequently adjusted by an intraspecies EF composed of toxicokinetic (EF_HK_) and toxicodynamic (EF_HD_) factors (US EPA, 2014). PBPK models can be used to compare internal dose metrics between the average population and potentially sensitive subpopulations (neonates, proton pump inhibitor [PPI] users, individuals with hypochlorhydria), and thus, the PBPK model was used to derive an EF_HK_. In the absence of any data with which to compare responses between average and sensitive individuals at comparable internal dose metrics, the EF_HD_ was set to a default value of 3. The RfD calculation is as follows:

$${\mathrm{POD}}_{\mathrm{HED}}=\left[\mathrm{POD}\;÷\;{\mathrm{EF}}_{\mathrm{AD}}\right]$$

$$\mathrm{RfD}={\mathrm{POD}}_{\mathrm{HED}}\;÷\;\left[{\mathrm{EF}}_{\mathrm{HK}}\;\times \;{\mathrm{EF}}_{\mathrm{HD}}\right]$$

where: RfD is mg kg^−1^ day^−1^; POD is expressed in terms of internal dose in rodents; HED is mg kg^−1^ day^−1^; EF_AD_ = EF for interspecies toxicodynamic variation (unitless); EF_HK_ = EF for intraspecies pharmacokinetic variation (unitless); EF_HD_ = EF for intraspecies toxicodynamic variation (unitless).

Because the oral cavity tumors in F344 rats were significantly elevated only at 180 ppm, and mechanistic data support thresholds in oral tissue response to Cr(VI) (see Section 3.1.1), an MOE analysis was conducted for these tumors. The MOE is defined as the ratio of the BMDL_10_ in an animal study to the estimated human exposure.

$$\mathrm{MOE}={\text{BMDL}}_{10\left(\text{animal}\right)}\;÷\;{\text{Exposure}}_{\left(\text{human}\right)}$$

where: MOE is unitless; BMDL_10(animal)_ = POD (mg kg^−1^ bodyweight of applied dose to rodent); Exposure_(human)_ = mean daily exposure (mg kg^−1^ bodyweight).

Human exposures can be either mean human exposures or high exposures (e.g., 95th percentile). MOE values ≥30 000 or ≥100 000 are considered by many to indicate low concern for human health (Barlow, Renwick, Kleiner, et al., 2006).

## 3 RESULTS

### 3.1 Dose–response analysis informed by mechanistic considerations

#### 3.1.1 Portal‐of‐entry effects

##### Oral mucosa

Exposure to Cr(VI) was associated with a relatively late onset of tumors in the oral cavity of male and female F344 rats (NTP, 2008) (Table 1). To date, no non‐neoplastic or pre‐neoplastic histopathological lesions that might serve as precursor events have been identified in the oral tissue of rats or mice exposed to up to 180 ppm Cr(VI) for 7 days, 13 weeks or 2 years (NTP, 2007, 2008; Thompson, Proctor, et al., 2011; Thompson et al., 2012). Toxicogenomic analyses indicate minimal, if any, gene expression changes in the oral mucosa of F344 rats or B6C3F1 mice exposed to ≤180 ppm Cr(VI) for 7 or 90 days (Thompson et al., 2016). Exposure to 180 ppm Cr(VI) for 28 days did not increase mutant frequency in oral tissue of Big Blue® F344 rats (Thompson, Young, et al., 2015). Taken together, these data indicate that Cr(VI) elicits minimal, if any, direct cellular responses in the oral mucosa of rats or mice.

In 2008, De Flora and colleagues proposed that the oral tumors in rats might have been the result of local irritation and oxidation by Cr(VI) at the highest dose—possibly combined with mechanical stimulation by water bottle cannulae (De Flora et al., 2008). Interestingly, we previously observed dose‐dependent decreases in the reduced/oxidized glutathione (GSH/GSSG) ratio (i.e., increased oxidation) in oral samples in F344 rats but not mice (Thompson et al., 2012). However, given the lack of gene expression changes in the oral mucosa (Thompson et al., 2016), the change in GSH/GSSG ratio may not have occurred in the oral mucosa tissue per se, but rather in the saliva or microbiota present in the oral cavity. We have also shown that high levels of Cr(VI) employed in the 2 year bioassay generally impaired the health of rodents, as indicated by reduced water intake, reduced bodyweight and iron deficiency (Suh et al., 2014). Taken together, these data indicate that the oral tumors in rats, which were significantly elevated only at 180 ppm Cr(VI), are unlikely to be initiated by direct contact. Moreover, the significant reduction in bodyweight gain suggests that rats exposed to 180 ppm exceeded a maximum tolerated dose, which, according to US EPA (2005) guidance, confounds the relevance of these tumors. Notably, 350 ppm was determined to be too toxic to use in the 2 year bioassay based on adverse effects observed in the 13 week study (NTP, 2008). Similarly, male mice only received ≤90 ppm Cr(VI) due to toxicity observed at 180 ppm in the 13 week study (NTP, 2008). Although general toxicity from achieving the maximum tolerated dose is not associated with oral tumors in F344 rats per se, rats do appear to have a proclivity toward oral cavity tumor development (NTP, 2008; Stout et al., 2009). Nevertheless, a number of mutagenic and non‐mutagenic chemicals induce squamous carcinomas in the oral cavity of rats (Greaves, 2012).

**Figure 2:**
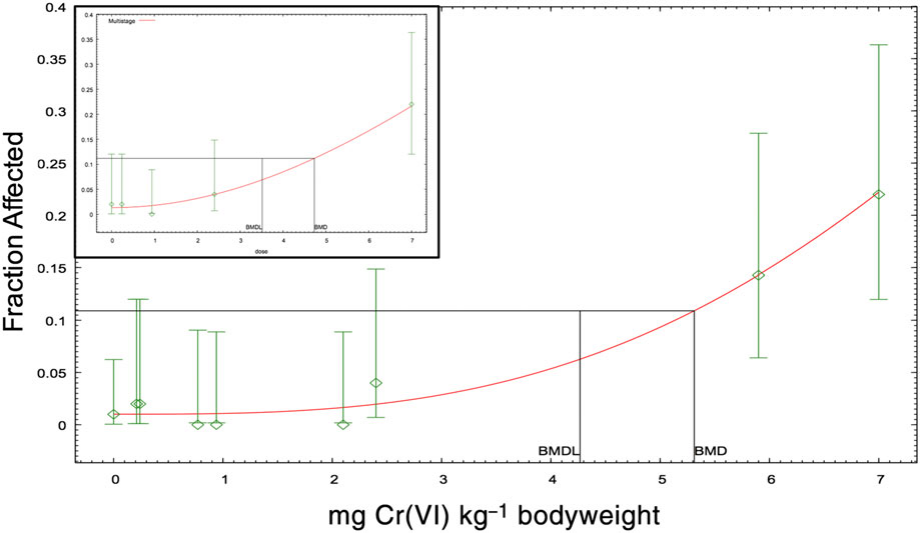
Dose–response modeling of oral tumor incidence in F344 rats. Tumor incidence in males and females combined (*P* = 0.78). (inset) tumor incidence in females only (*P* = 0.75). Both curves relflect predictions of the multistage model. Data adapted from NTP (2008). BMD, benchmark dose; BMDL, benchmark dose (with corresponding 95% lower confidence limit) [Colour figure can be viewed at wileyonlinelibrary.com]

Oral tumors were significantly elevated (14% in males, 22% in females) in the highest treatment group (Table 1). With this highest dose group included, the Cochran–Armitage trend test in the US EPA's BMD software is statistically significant for each sex; however, the trend test is not significant when the highest dose group is removed. This indicates that there is no statistical evidence of a dose–response up to 60 ppm Cr(VI) (OECD, 2006). Because the PBPK model for Cr(VI) does not predict Cr(VI) flux or total chromium levels in the oral mucosa, and because the data indicate that chromium does not act directly on the oral mucosa, mg kg^−1^ bodyweight was selected as the dose metric to model the oral tumor incidence in rats. Per US EPA guidance (US EPA, 2005, 2012), a BMR of 10% extra risk was selected to model tumor incidence. The BMDL_10_ values for males, females and both sexes combined were 4.3, 3.5 and 4.3 mg Cr(VI) kg^−1^ bodyweight, respectively. The plots for the combined analysis and females only are shown in Figure 2. Consistent with the Cochran–Armitage trend test (above), both plots for oral tumors indicate a non‐linear dose–response.

These plots, together with the mechanistic data above, indicate that a cancer slope factor, for a linear exposure–response, based on oral tumors is unwarranted. Instead, we present an MOE analysis. A preliminary analysis of drinking‐water data in the USA indicate mean and 95th percentile concentrations of Cr(VI) of 0.001 and 0.003 ppm, respectively (US EPA, 2017). Therefore, an 80 kg adult consuming 2.5 liters of water per day that contains 0.001–0.003 ppm Cr(VI) would receive doses ranging from 3.1E‐5 to 9.4E 5 mg kg^−1^ day^−1^. Dividing these exposure estimates into the BMDL_10_ of 3.5 mg kg^−1^ day^−1^ results in MOE values greater than 100 000 and 30 000, respectively. Such large MOE values are considered by many to indicate a low concern for risk to human health (Barlow et al., 2006).

##### Tumors of the small intestine

**Figure 3:**
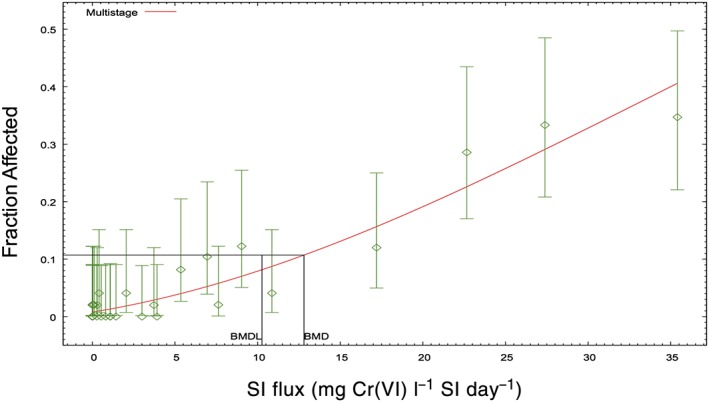
Dose–response modeling of intestinal tumor incidence in B6C3F1 mice. Combined incidence of adenomas and carcinomas in male and female mice in the duodenum, jejunum and ileum (multistage model; *P* = 0.2644). BMDL_10_ values are shown in Table 3. Data adapted from NTP (2008). BMD, benchmark dose; BMDL, benchmark dose (with corresponding 95% lower confidence limit); SI, small intestine [Colour figure can be viewed at wileyonlinelibrary.com]

Table 2 lists the incidence of intestinal adenomas, carcinomas, and adenomas and carcinomas combined, as well as diffuse epithelial hyperplasia in mice as a function of SI sectional Cr(VI) flux. The flux of chromium is highest in the proximal SI (duodenum) and lowest in the distal intestine (ileum); the incidences of hyperplasia and tumor decrease as flux decreases. Statistical analyses (see Methods, Section 2.2) indicated that the incidence of intestinal tumors did not differ between male and female mice in any intestinal segment. Neither the main effect (χ^2^(6) = 6.79, *P* = 0.34) nor the interaction (χ^2^(12) = 14.09, *P =* 0.30) of sex was significant, indicating that these data could be modeled together. As a demonstration of how well the intestinal flux estimates predict response in the mouse SI, Figure 3 shows a dose–response for the combined incidence of adenomas or carcinomas in each intestinal section of male and female mice as a function of flux. The BMD_10_ and BMDL_10_ values were 12.8 and 10.3 mg Cr(VI) l^−1^ day^−1^ (Table 3). Because intestinal tumors are thought to progress from adenomas to carcinomas (Brix, Hardisty, & McConnell, 2010; Greaves, 2012; McConnell, Solleveld, Swenberg, & Boorman, 1986), adenomas and carcinomas were also modeled separately. As expected, the BMDL_10_ for carcinomas (28.1 mg Cr(VI) l^−1^ day^−1^) was higher than adenomas (13.2 mg Cr(VI) l^−1^ day^−1^) (Table 3).

**Table 3:** Summary of BMD modeling of intestinal lesions based on internal dose^a^

Endpoint	Segment	BMD_10‐flux_	BMDL_10‐flux_	*P* value	Model	Groups^b^
Adenoma/carcinoma combined	D, J, I	12.81	10.26	0.2644	multistage	30
Adenomas	D, J, I	15.49	13.15	0.2722	multistage	30
Carcinomas	D, J, I	35.46	28.12	0.2265	multistage	30
DEH	D, I	2.10	1.68	0.0679	Hill	20
DEH (drop 3 highest groups)	D, I	2.13	1.70	0.0986	Hill/log‐logistic	17
DEH (omit 7.6 group)	D, I	2.06	1.70	0.1992	Hill	19

BMD, benchmark dose; BMDL, benchmark dose (with corresponding 95% lower confidence limit); D, duodenum; J, jejunum; I, ileum; DEH, diffuse epithelial hyperplasia.

a SI flux (mg Cr(VI) l^−1^ SI day^−1^).

b No. of dose groups = 2 sexes × no. of segments × no. of doses.

##### Diffuse epithelial hyperplasia of the small intestine

Exposure to high levels of Cr(VI) induces diffuse epithelial hyperplasia in the duodenum and, to a lesser extent, the jejunum in mice (Table 2). As with tumors, the incidence of diffuse epithelial hyperplasia was highest in the proximal SI (duodenum) and lowest in the distal intestine (ileum). Diffuse epithelial hyperplasia is a non‐neoplastic lesion that, under chronic wounding, can lead to increased stem cell proliferation, which can promote transformation and carcinogenesis (Cohen & Ellwein, 1990; Cohen, Gordon, Singh, Arce, & Nyska, 2010; Tomasetti & Vogelstein, 2015). Because Cr(VI) flux through intestinal sections well describes the tumor response in the SI, and hyperplasia is a critical preceding event to tumor formation, intestinal flux was used to model hyperplasia incidence from the NTP (2008) 2 year bioassay. As described above, statistical analyses were conducted to determine whether male and female diffuse epithelial hyperplasia could be modeled together. The overall incidence of hyperplasia did not differ significantly between female and male animals in any of the intestinal segments.

**Figure 4:**
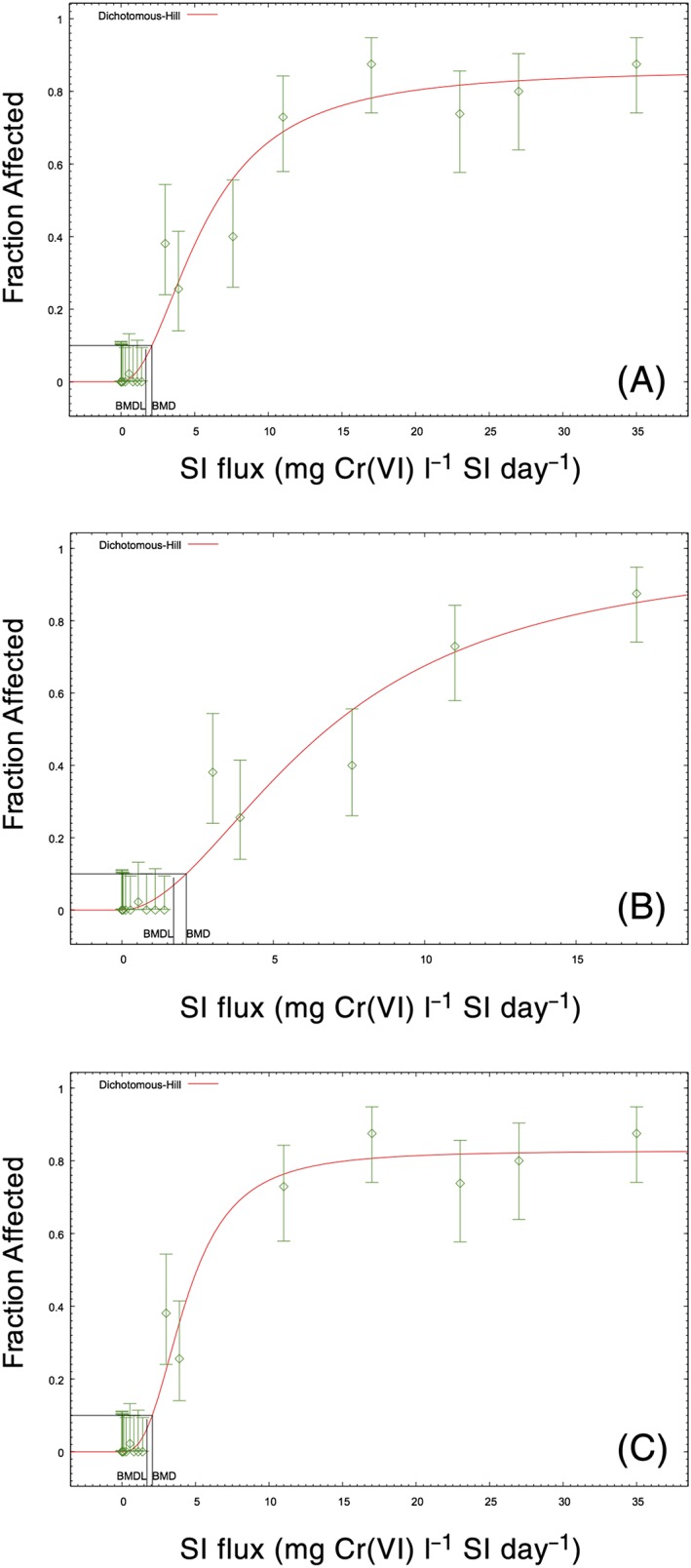
Dose–response modeling of the diffuse epithelial hyperplasia incidence in B6C3F1 mice. (A) Combined diffuse epithelial hyperplasia incidence in the duodenum and ileum of male and female mice (Hill; *P* = 0.0679). (B) Diffuse epithelial hyperplasia after dropping the three highest dose groups in the response plateau (Hill and log‐logistic; *P* = 0.0986). (C) Diffuse epithelial hyperplasia after omitting single dose at 7.6 SI flux units (Hill; *P* = 0.1992). All three models result in BMD_10_ and BMDL_10_ values of 2.1 and 1.7 SI flux units (mg Cr(VI) l^−1^ SI) (Table 3). Incidence data are adapted from NTP (2008). BMD, benchmark dose; BMDL, benchmark dose (with corresponding 95% lower confidence limit); SI, small intestine [Colour figure can be viewed at wileyonlinelibrary.com]

As discussed in Methods, Section 2.2, the jejunum data were not modeled, due to uncertainties introduced by the experimental protocol for diagnosing hyperplasia. Including these data would shift the dose–response curve rightward, thereby resulting in a less conservative BMDL (see Thompson et al., 2014). Figure 4(A) shows the best fitting model of the combined male and female duodenum and ileum data. The *P* value for global fit (0.067) was below the US EPA's preferred target minimum of 0.1, but was higher than the minimum acceptable for the multistage model. Removing the three highest dose groups increased the *P* value to 0.1 (rounded from 0.098) (Figure 4B). Examination of Figure 4(A) and the scaled residuals indicates that data points at doses of 3.0 and 7.6 flux units were penalizing the *P* value. Removing the 7.6 dose group increased the *P* value to 0.2 (rounded from 0.199). All three modeling approaches (using all data, dropping three highest dose groups, or omitting one potential outlier group at an SI flux of 7.6 mg Cr(VI)l^−1^ SI day^−1^) resulted in BMD_10_ and BMDL_10_ values of 2.1 and 1.7 mg Cr(VI) l^−1^ day^−1^ (Table 3). Modeling duodenum and ileum hyperplasia data in males and females separately resulted in BMDL_10_ values of 1.6 and 2.2 mg Cr(VI) l^−1^ day^−1^, respectively (data not shown). Because BMD modeling was conducted on 20 data points, representing 869 observations (5 dose groups × 2 sexes × 2 intestinal segments × ~45 animals per group), diffuse epithelial hyperplasia was also modeled using a 5% BMR. The BMDL_5_ values were all within the range of observed data. The three modeling approaches resulted in BMDL_5_ values ranging from 1.1 to 1.2 mg Cr(VI) l^−1^ day^−1^. The BMDL_5_ values for males and females separately were 0.93 and 2.1 mg Cr(VI) l^−1^ day^−1^, respectively (data not shown). The minor differences of modeling sexes individually argue in support of using the entire data set.

**Figure 5:**
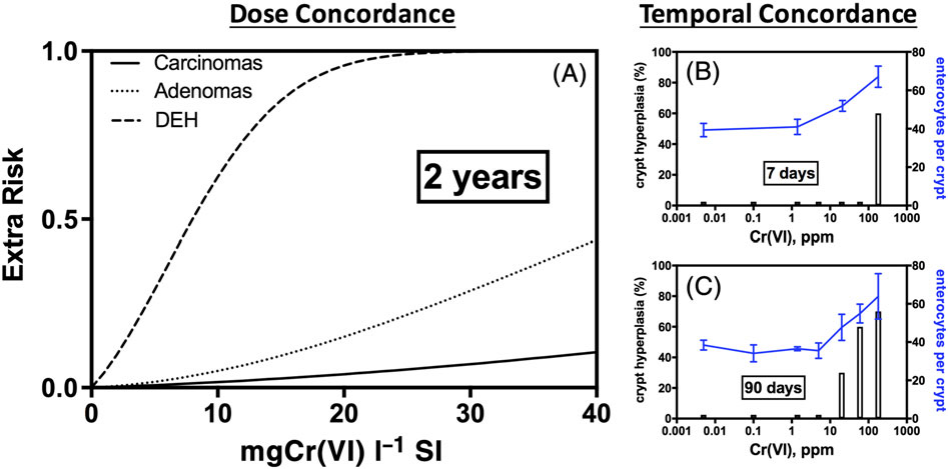
Dose and temporal concordance of intestinal diffuse epithelial hyperplasia, adenomas and carcinomas in mice. (A) Dose concordance (based on Cr(VI) SI flux): As evidenced by multistage models from the US EPA's benchmark dose software v.2.6 for three endpoints using data from male and female mice. Incidence data are adapted from NTP (2008). Tumor responses are based on all three intestinal sections from male and female mice. DEH responses are based on duodenum and ileum data from male and female mice. These models are not used for quantitative dose–response analysis, but rather serve to compare visually the responses progressing from non‐neoplastic hyperplasia to adenomas and carcinomas. (B) Temporal concordance (7 days of exposure): As evidenced by crypt hyperplasia in female mice in the absence of neoplastic lesions. Bars represent incidence in hematoxylin and eosin‐stained sections (Thompson, Proctor, et al., 2011). Note: The short bars indicate empirical observations with 0% incidence. Blue line represents mean ± SD for counted cells in a second study (Thompson, Wolf, et al., 2015). (C) Temporal concordance (90 days of exposure): As evidenced by crypt hyperplasia in female mice in the absence of neoplastic lesions. Bars represent incidence in hematoxylin and eosin‐stained sections (Thompson, Proctor, et al., 2011). Note: The short bars indicate empirical observations with 0% incidence. Blue line represents mean ± SD for counted cells (O'Brien et al., 2013). DEH, diffuse epithelial hyperplasia; SI, small intestine

As noted previously, diffuse epithelial hyperplasia has been considered an early precursor key event in the MOA for intestinal carcinogenesis (Becker et al., 2015; HealthCanada, 2015; TCEQ, 2016; Thompson et al., 2013; Thompson et al., 2014). As would be expected for a precursor effect, the incidence of hyperplasia clearly increases at doses that are lower than those associated with increased incidence of tumors (Figure 5A). Hyperplasia also preceded tumorigenesis temporally, because Cr(VI) has been shown to increase cell proliferation in mice (without neoplastic or pre‐neoplastic lesions) after 7 and 90 days of exposure (Figure 5B,C) (NTP, 2007; O'Brien et al., 2013; Thompson, Proctor, et al., 2011; Thompson, Wolf, et al., 2015).

##### Histiocytic cellular infiltration of mesenteric lymph nodes

Histiocytic cellular infiltration was observed in the duodenum and mesenteric lymph nodes of mice and rats in the NTP 13‐week and 2‐year bioassays (NTP, 2007, 2008). In the 13‐week study, histiocytic infiltration was noted in the mouse duodenum at lower doses than in the mesenteric lymph nodes. As discussed in NTP (2007), several “compounds are thought to initially induce infiltration of macrophages into the lamina propria of the small intestine and subsequently histiocytosis in the mesenteric lymph node.” Oral exposure to many compounds can induce “accumulation enteropathies,” where histiocytes in the SI ingest foreign material and subsequently infiltrate the villous lamina propria and mesenteric lymph nodes—often showing similar accumulation of foreign bodies in macrophages of both regions (Gopinath, Prentice, & Lewis, 1987). It is thought that mesenteric lymph nodes act as a “storage depot” for macrophages that are unable to degrade ingested cellular contents (Gopinath et al., 1987). The 13‐week and 2‐year NTP Cr(VI) study reports noted the similarity in histology between the histiocytes in the intestine and mesenteric lymph nodes (NTP, 2007, 2008). X‐ray fluorescence microscopy indicates chromium localization to intestinal villi (not crypts) (Thompson, Seiter, et al., 2015; Thompson, Wolf, et al., 2015), and thus provides evidence for accumulation enteropathy. In the US EPA (2010) draft assessment of Cr(VI), histiocytic infiltration into the duodenum was not modeled in either species, whereas histiocytic infiltration of mesenteric lymph nodes was modeled in male and female mice, but not rats. As it is likely that histiocytic infiltration of mesenteric lymph nodes is a consequence of effects in the duodenum, we consider this an adaptive response to the presence of foreign material (i.e., chromium) and, therefore, it was not considered relevant for RfD derivation. It is reasonable to conclude that protection against intestinal injury (e.g., diffuse epithelial hyperplasia) will mitigate histiocytic infiltration into mesenteric lymph nodes.

#### 3.1.2 Systemic effects

As mentioned previously, no adverse effects were observed in the 2‐year bioassay on Cr(III) (NTP, 2010). Therefore, adverse systemic effects of Cr(VI) are potentially due to (1) direct effects of Cr(VI) in the blood, or (2) secondary effects such as changes in blood redox or iron homeostasis. Exposure to Cr(VI) was shown previously to alter serum GSH/GSSG levels and ratios (Thompson, Proctor, et al., 2011; Thompson et al., 2012), as well as induce iron depletion (Suh et al., 2014). Because the Cr(VI) PBPK model (Kirman et al., 2017) can estimate the amount of Cr(VI) entering (i.e., fluxing) into the portal circulation from the gastrointestinal tract, this dose metric was used for effects of Cr(VI) manifested beyond the intestinal mucosa, whether by direct or indirect mechanisms.

##### Liver

**Figure 6:**
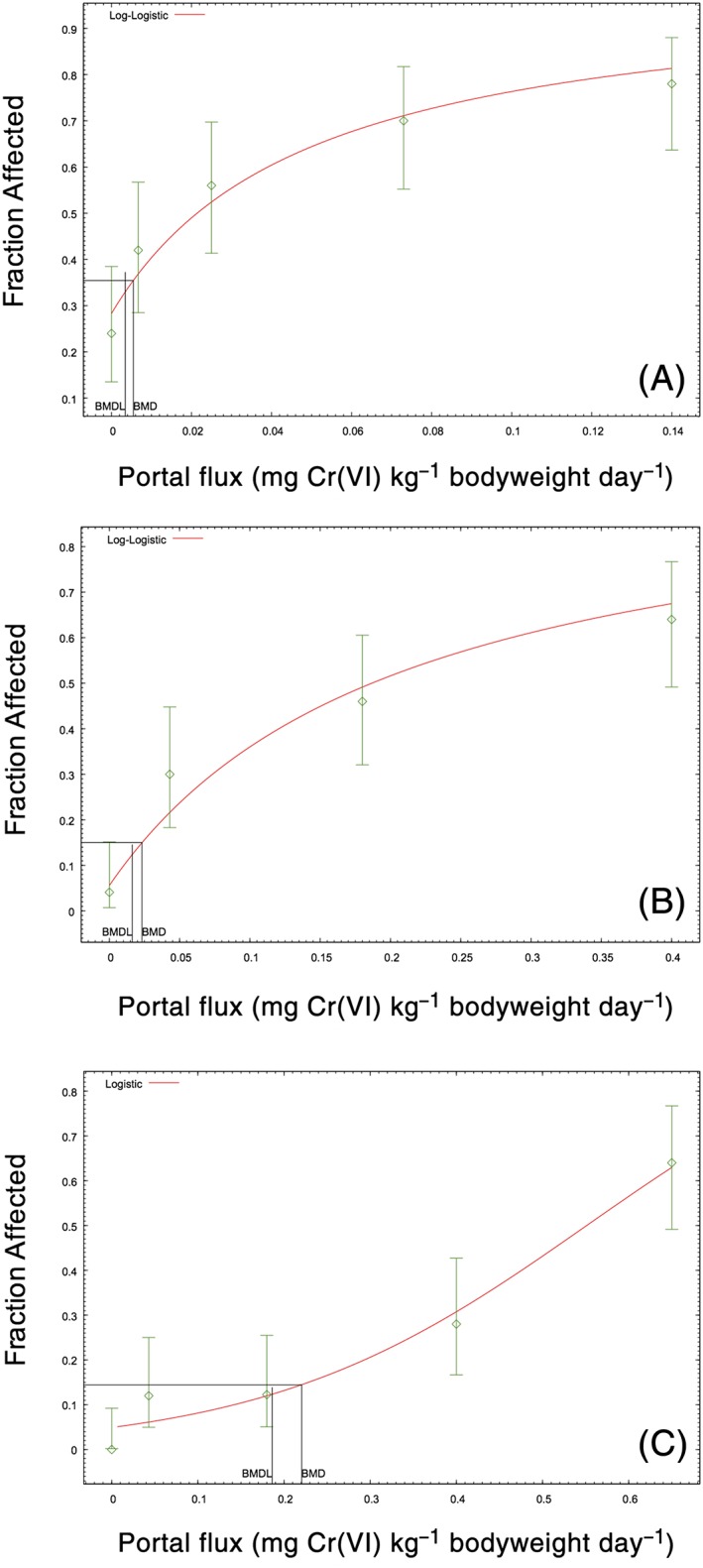
Dose–response modeling of the systemic effects. (A) BMD plot of incidence of chronic liver inflammation in female rats (*P* = 0.64). (B) BMD plot of incidence of histiocytic cellular infiltration into the liver of female mice (*P* = 0.25). (C) BMD plot of cytoplasmic alteration of the acinus pancreas in female mice (*P* = 0.13). BMDL_10_ values are shown in Table 4. Incidence data are adapted from NTP (2008). BMD, benchmark dose; BMDL, benchmark dose (with corresponding 95% lower confidence limit) [Colour figure can be viewed at wileyonlinelibrary.com]

The incidence of chronic liver inflammation was significantly elevated in female rats (Table 1), but not in male rats or mice. According to the NTP (2008) study authors, “[C]hronic inflammation is consistent with changes that are considered to be background or spontaneous lesions commonly observed in aged rats and appear to be exacerbated by exposure.” Notably, this effect was not listed in the summary table of the NTP (2008) cancer bioassay. These uncertainties limit the utility of this endpoint for RfD derivation; however, the endpoint is analyzed herein because it has served as the basis for toxicity criteria set in California (OEHHA, 2011). Similarly, histiocytic cellular infiltration was significantly elevated in female mice (Table 1), but not male mice. Although listed in the summary table of NTP (2008), the relevance of the lesions was considered by the NTP study authors to be unknown. As with the histiocytic infiltration into the duodenum and mesenteric lymph nodes, these infiltrates may be present to scavenge chromium. Indeed, the NTP study authors described infiltration into the liver as possible evidence of “phagocytosis of some insoluble chemical precipitate.” Despite the questionable relevance of these liver effects, BMD modeling was conducted for comparison to adverse effects in the SI (Figure 6A,B; Table 4).

**Table 4:** Summary of BMD modeling of systemic effects (Cr(VI) portal flux)

Endpoint	Species	Sex	BMD_10‐flux_	BMDL_10‐flux_	*P* value	Model^a^
Chronic liver inflammation	Rats	F	0.0055	0.0034	0.6434	Log‐logistic
Histiocytic cellular infiltration, liver	Mice	F	0.023	0.016	0.2522	Log‐logistic^b^
Cytoplasmic alteration of acinus pancreas	Mice	F	0.22	0.19	0.1265	Logistic
Cytoplasmic alteration of acinus pancreas	Mice	M	0.24	0.17	0.516	Multistage

BMD, benchmark dose; BMDL, benchmark dose (with corresponding 95% lower confidence limit); F, female; M, male.

a Model with lowest Akaike information criterion.

b Dropped highest dose group to improve model fit.

Portal flux (mg Cr(VI) kg^−1^ bodyweight day^−1^).

##### Pancreas

US EPA (2010) also modeled cytoplasmic alteration of the acinus pancreas of female mice, even though it was not included in the summary of non‐neoplastic lesions in NTP (2008). According to the NTP (2008) study authors, cytoplasmic alteration was “characterized by depletion of cytoplasmic zymogen granules from the pancreatic acinar epithelial cells.” Loss of zymogen (degranulation) is said to represent a physiological feature rather than a pathological process (Gopinath et al., 1987). Such lesions are observed in rats treated with diuretics (likely related to dehydration), as well as those in conditions of food deprivation (Gopinath et al., 1987). As noted by the NTP (2008) study authors, the significance of these lesions is unknown. Water consumption rates in the two highest male and female dose groups were less than controls throughout the study. In the second year of the study, the average water consumption was reduced by 15% and 35% in the two highest male dose groups and by 25% and 32% in the two highest female dose groups (NTP, 2008). It is therefore conceivable that the effects in the pancreas might be due to indirect mechanisms, such as reduced water intake due to poor palatability. Despite the questionable significance of pancreatic alterations, these lesions were modeled in both male and female mice using portal flux. The BMD plot for female mice is shown in Figure 6(C), and the BMDL_10_ values for males and females are listed in Table 4.

##### Reproductive and developmental toxicity

The effects of Cr(VI) on reproductive and developmental toxicity were determined previously by the US EPA to occur at higher exposure doses than effects in the NTP (2008) chronic bioassay and, thus, no reproductive or developmental toxicity effects were carried forward for dose–response analysis (US EPA, 2010). Consistent with this conclusion, other regulators have proposed oral toxicity criteria based on portal‐of‐entry effects, as opposed to reproductive and developmental toxicity (HealthCanada, 2015; TCEQ, 2016). In contrast, OEHHA (2010) developed a maximum allowable dose level for female reproductive toxicity in Swiss albino mice (Murthy, Junaid, & Saxena, 1996). The maximum allowable dose level was based on a NOAEL; however, US EPA (2010) concluded that NOAEL/low‐observable‐adverse‐effect‐level values could not be identified in Murthy et al. (1996) due to inadequate reporting of data. In brief, Murthy et al. (1996) exposed Swiss albino mice to 250, 500 and 750 ppm Cr(VI) in drinking water for 20 days, and reported ovarian effects (decreases in the number of follicles) in mice exposed to 250 ppm. In the same study, mice in another group were exposed to 0.05, 0.5 and 5 ppm Cr(VI) for 90 days. Unlike the 20 day study, no quantitative data were provided; rather, it was reported that, using electron microscopy, there were disintegrated membranes in follicular cells of the 5 ppm group (Murthy et al., 1996). OEHHA selected 0.5 ppm as the study NOAEL, which they determined to be equivalent to 0.142 mg kg^−1^ (OEHHA, 2010) using dose estimates from another study. Importantly, Murthy et al. neither provide estimates of dose, nor of bodyweight and water intake data needed to estimate dose. Moreover, Murthy et al. do not mention whether Cr(VI) concentrations were analytically verified. To this non‐statistically based NOAEL, consistent with the requirements of Proposition 65, OEHHA applied a 1000‐fold safety factor. It should be noted that chromium levels were not significantly elevated in the plasma, erythrocytes or livers (a proxy for systemic chromium) of female mice and rats exposed to ≤1.4 ppm for 90 days (Kirman et al., 2012; Thompson, Proctor, et al., 2011; Supporting information Figure S1). Similarly, NTP reported no significant increases in plasma or erythrocyte chromium levels in female mice exposed to 5 ppm Cr(VI) for 6, 13, 182 or 371 days (NTP, 2008). It is therefore unlikely that low Cr(VI) levels in drinking water pose a direct risk to ovarian follicles. As previously mentioned, US EPA (2010) did not derive a POD from Murthy et al. (1996), due in part to inadequate reporting in the study.

The NTP has conducted several studies that inform the potential for reproductive and developmental toxicity effects from Cr(VI) exposure. No significant microscopic lesions were observed in ovaries of F344 rats (mice not examined) exposed to ≤350 ppm in the NTP (2007) 90‐day drinking water study, nor were such lesions observed in earlier feed studies in mice and rats (NTP, 1996a, 1996b). No effects on ovary weight or reproductive performance were observed in F_0_ or F_1_ BALB/c mice (NTP, 1997). Testis weight in F344 rats was unaffected by exposure to ≤350 ppm Cr(VI) for 13 weeks (NTP, 2007). Similarly, testis weights in B6C3F1 and BALB/c mice were unaffected by exposure to ≤350 ppm Cr(VI) for 13 weeks; however, testis weight was reduced 11% in am3‐C57BL/6 mice, which was attributed to a 36% decrease in bodyweight (NTP, 2007). Earlier feed studies with Cr(VI) also found no effects on testis weight in rats or mice (NTP, 1996a, 1996b, 1997).

Many of the earlier reproductive drinking water studies employed very high concentrations and often very few doses. Moreover, the studies are ambiguous as to whether the concentrations are reported in terms of the Cr(VI) ion or the Cr(VI) salt (e.g., potassium dichromate). The studies also failed to mention (and likely to conduct) dose formulation analysis to confirm Cr(VI) concentrations. Overall, the concentrations in these studies were much higher than those in the NTP (2008) cancer bioassay (Supporting information Figure S2), and adverse effects therefore occurred at higher concentrations than effects in the cancer bioassay. Similarly, many of the developmental toxicity studies for Cr(VI) employ higher concentrations than in the NTP (2008) cancer bioassay (Supporting information Figure S2), and adverse effects were observed at very high doses. As mentioned previously, no reproductive or developmental effects were carried forth as potential RfD values in US EPA (2010).

Since the release of the US EPA (2010) draft assessment, several reproductive and developmental toxicity studies have been published. None of these studies appear to have followed any regulatory guidelines, such as OECD 422. Many of the studies lack mg kg^−1^ dose estimates or data to estimate such (e.g., maternal bodyweight and water intake). Many of the studies are ambiguous as to whether the concentrations are in terms of Cr(VI) ion or compound, and none report having analytically verified the Cr(VI) dose formulation. Most of the reported effects occurred at high Cr(VI) concentrations, consistent with earlier studies (see above). The Cr(VI) concentrations employed in these studies are higher than those in the NTP (2008) cancer bioassay (Supporting information Figure S2).

A few recent studies claim that high concentrations of Cr(VI) disrupt endocrine function and, therefore, label Cr(VI) as an “endocrine disruptor” (Banu et al., 2017; Stanley et al., 2013). We therefore queried US EPA's Tox21 consortium database to determine whether data were available to support the notion of Cr(VI) as an endocrine disrupter. There was no significant indication of androgen, estrogen, or thyroid receptor activation/binding (see Appendix B). Nevertheless, these data do not preclude the possibility that high concentrations of Cr(VI) disrupt endocrine function indirectly (e.g., oxidative stress, iron perturbation). Based on these considerations, reproductive and developmental toxicity were not considered further for RfD development.

### 3.2 Reference dose derivation

Based on BMD modeling of non‐neoplastic lesions (Tables 3 and 4), five data sets were carried forward for RfD derivation: diffuse epithelial hyperplasia (combined analysis in male and female mice), chronic liver inflammation in female rats, histiocytic cellular infiltration in the liver of female mice, and cytoplasmic alteration of the acinus pancreas in male and female mice.

#### 3.2.1 Interspecies extrapolation

US EPA guidance indicates that the interspecies EF should consist of toxicokinetic (EF_AK_) and toxicodynamic (EF_AD_) factors (US EPA, 2014). Per US EPA, PBPK models can obviate the need for EF_AK_, because the HED is computed directly via the PBPK model. The use of PBPK models for target tissue and portal flux of Cr(VI) therefore obviates the need for an EF_AK_. In the absence of any data to which responses to Cr(VI) in target tissues could be compared across species at comparable dose metrics, a default threefold EF_AD_ was applied. This factor was applied at the point where the extrapolation was made (i.e., the rodent internal dose metric) rather than applying it in the final step of RfD derivation. After each rodent BMDL was reduced by threefold, the human PBPK model was used to predict the applied dose (POD_HED_) that results in the adjusted internal dose metric.

#### 3.2.2 Intraspecies extrapolation

**Figure 7:**
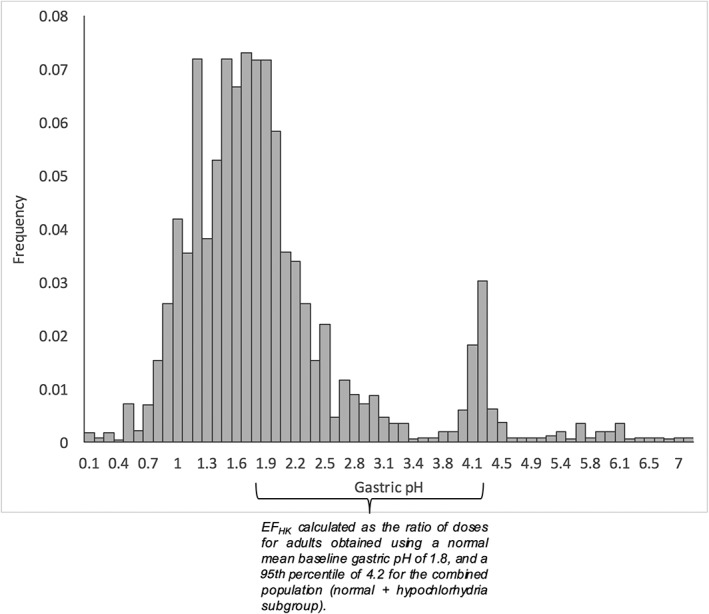
Basis for EF_HK_. Bimodal distribution for baseline gastric pH in humans. Data adapted from Ayazi et al. (2009)

The POD_HED_ values derived using the human PBPK model include contributions of various life stages (neonate, child, youth, adult, elderly), and therefore already address several important sources of variation as a function of age that may contribute to increased risk (e.g., higher baseline gastric pH in neonates). This assessment focuses on characterizing variation in baseline gastric pH, a key model parameter that determines the delivery of Cr(VI) to the SI, and subsequent risk, using published gastric pH data (Ayazi et al., 2009). Ayazi et al. reported a bimodal distribution for gastric pH, with the normal subgroup distributed about a median pH of 1.7–1.8, and a hypochlorhydria subgroup with a median pH of approximately 4.2 (Figure 7). The 95th percentile for the combined distribution (normal and hypochlorhydria) also corresponds to a gastric pH of approximately 4.2. Using these data, a value for EF_HK_ was calculated using a ratio of doses:

$${\mathrm{EF}}_{\mathrm{HK}}=\left[{\mathrm{POD}}_{\mathrm{HED}}\;\mathrm{at}\;\mathrm{pH}=4.2\right]/\left[{\mathrm{POD}}_{\mathrm{HED}}\;\mathrm{at}\;\mathrm{pH}\;1.8\right]$$

Consideration was also given to using PPI users as the basis for POD in the numerator of the above equation. However, this approach resulted in slightly lower values of EF_HK_ than the use of a baseline pH of 4.2 (data not shown). The value of EF_HK_ is dose‐dependent due to non‐linear toxicokinetics (i.e., depletion of gastric reducing agents). Therefore, EF_HK_ values are determined for the POD_HED_ values for each toxicity endpoint (Table 5). In the absence of any human data to which responses to Cr(VI) in target tissues could be compared at comparable dose metrics, a default threefold EF_HD_ was applied.

**Table 5:** Candidate RfD values

Effect	Species	Sex	POD_HED_ (mg kg^−1^ day^−1^)^a^	EF_HK_	EF_HD_	RfD^b^ (mg kg^−1^ day^−1^)
Diffuse epithelial hyperplasia	Mouse	M, F	0.028	2.3	3	4.0E‐3
Diffuse epithelial hyperplasia (5% BMR)^c^	Mouse	M, F	0.020	2.4	3	3.0E‐3
Chronic liver inflammation	Rat	F	0.023	2.4	3	3.0E‐3
Histiocytic cellular infiltration into liver	Mouse	F	0.022	2.4	3	3.0E‐3
Cytoplasmic alteration of the acinus pancreas	Mouse	F	0.11	1.7	3	2.0E‐2
Cytoplasmic alteration of the acinus pancreas	Mouse	M	0.10	1.8	3	2.0E‐2

BMR, benchmark response; RfD, reference dose.

a POD_HED_ already has a threefold EF_AD_ applied.

b All RfDs are based on 10% BMR unless otherwise noted (and rounded to 1 significant figure).

c BMR of 5% was selected for this endpoint due to the robust data set (see text).

#### 3.2.3 Reference dose selection

**Figure 8:**
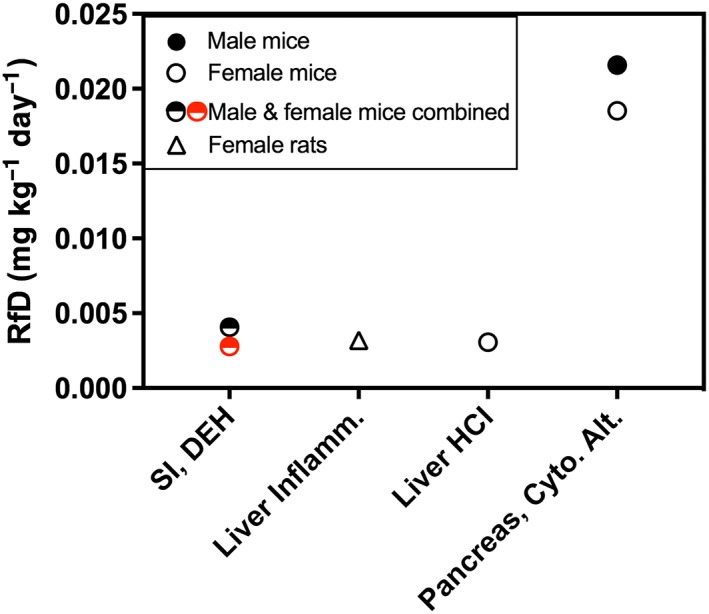
Candidate RfD array. Open and closed circles represent female and male mice, respectively. Half‐filled circles represent male and female data modeled together. Open triangles represent female rat data. Red and black symbols are based on benchmark response values of 5% and 10% extra risk, respectively. Cyto. Alt., cytoplasmic alteration; D, duodenum; DEH, diffuse epithelial hyperplasia; HCI, histiocytic cellular infiltration; Inflamm., inflammation; RfD, reference dose; SI, small intestine

Candidate RfD values are shown in Figure 8. Effects in the pancreas resulted in the highest candidate RfD values of 0.02 mg kg^−1^ day^−1^ (Table 5). Liver effects in both rats and mice resulted in candidate RfD values of 0.003 mg kg^−1^ day^−1^. The candidate RfD values for diffuse epithelial hyperplasia in males and females were 0.003 and 0.004 mg kg^−1^ day^−1^ based on 5% and 10% BMR values, respectively. Because diffuse epithelial hyperplasia is an early key event in the MOA for intestinal tumors in mice, we selected this as the basis for the RfD. Using the more conservative 5% BMR, we selected the RfD of 0.003 mg kg^−1^ day^−1^ based on diffuse epithelial hyperplasia. This RfD protects against other non‐cancer effects, as well as carcinogenesis in the gastrointestinal tract.

## 4 DISCUSSION

Analyses presented herein provide dose–response evidence that diffuse epithelial hyperplasia precedes intestinal carcinogenesis in both dose and time. These findings are consistent with mechanistic evidence for the early induction of hyperplasia (after only 1 week of exposure) and lack of in vivo genotoxic responses. The US EPA supports RfD derivation for carcinogens when the MOA can reasonably be concluded to occur through non‐linear mechanisms (US EPA, 2005). As such, the RfD of 0.003 mg kg^−1^ day^−1^ is protective against both intestinal wounding and intestinal carcinogenesis induced by Cr(VI). Such threshold‐based toxicity criteria have been developed for the SI carcinogens captan and folpet based on evidence for a similar MOA involving intestinal wounding and chronic regenerative hyperplasia (EFSA, 2009; Gordon, 2007; US EPA, 2004).

The RfD herein is approximately two‐fold lower than the RfD we derived previously for Cr(VI) (Thompson et al., 2014). The differences in RfD values arise from offsetting factors such as the revised incidence data (see Section 2.2.3), dropping of high doses in the previous RfD BMD modeling and revisions to the PBPK models. Because our previous BMDL_5_ (0.84 mg Cr(VI) l^−1^ SI day^−1^) is similar to the 1.1 mg Cr(VI) l^−1^ SI day^−1^ BMDL_5_ derived herein, and the EF values applied herein are similar to the uncertainty factors applied in our previous RfD, the main reason for the twofold decrease in the RfD stems from the revised human PBPK model (Kirman et al., 2017), which contains three reduction pools rather than one in the earlier models (Kirman et al., 2012; Kirman et al., 2013). Data indicate a low capacity/fast reduction pool, a higher capacity/slower reduction pool and a high capacity/slow reduction pool. The estimated capacities for the fast reduction pools in humans is 0.68–2.6 mg l^−1^ (depending on whether fed or fasted), 6.1 mg l^−1^ in mice and 7.1 mg l^−1^ in rats. These data indicate that depletion of the fast pool occurs at lower Cr(VI) doses in humans than in either mice or rats. Nevertheless, these pools, which are replenished between bouts of exposure, are sufficient for reducing environmental levels of Cr(VI) that are typically ≤0.003 mg l^−1^.

The RfD proposed herein is health protective for most individuals. For example, Cr(VI) reduction is pH‐dependent, and therefore, life stage differences in gastric pH are accounted for in the PBPK model. Life stage differences in water and food intake are also accounted for in the PBPK model. Because there are fast, medium and slow reduction pools that each have different capacities, the simultaneous accounting for life stage differences in intake and gastric pH are factored into the estimation of safe human doses. In addition to life stage, the human PBPK model was used to address human variation by considering Cr(VI) reduction in individuals with high gastric pH due to use of medication such as PPIs, or those with hypochlorhydria. Quantitative differences in dose were used to support the pharmacokinetic human variability EF (i.e., EF_HK_).

The RfD proposed herein is identical to the current EPA RfD that is based on a NOAEL from a 1‐year bioassay in rats exposed to ≤25 ppm Cr(VI) (Mackenzie et al., 1958). In that study, no adverse effects were observed in rats exposed to up to 25 ppm Cr(VI) in drinking water. EPA characterizes the confidence in their RfD as low because of the “small number of animals tested, the small number of parameters measured, and the lack of toxic effect at the highest dose tested.” This “low” confidence in the existing RfD based on Mackenzie et al. (1958) does not mean that the value is not health protective, but rather that the scientific basis of the RfD could be improved. This is reflected by EPA's adjustment of the NOAEL by a 1000‐fold uncertainty factor. Our proposed RfD is based on considerably more scientific information, including data from a 2‐year bioassay, rodent PBPK models developed using target tissue and gastric reduction data, human PBPK models informed by human pharmacokinetic data, quantitative dose–response modeling and MOA research. The uncertainties associated with potential pharmacodynamic differences across species and individuals are each addressed with default EF_AD_ and EF_HD_ values of 3 each. These EFs are akin to the threefold interspecies and intraspecies uncertainty factors (UF_A_ and UF_H_) factors often applied to account for pharmacodynamic uncertainties. As such, only a 10‐fold uncertainty is applied in the proposed RfD, as compared to the 1000‐fold uncertainty in the current EPA RfD. Although the RfD herein is identical to that listed in EPA's Integrated Risk Information System (IRIS), the scientific basis of our value is greater than the IRIS value and therefore we characterize the confidence in our RfD as “high.”

In conclusion, we have derived several candidate RfD values for Cr(VI) using sophisticated risk assessment approaches that greatly improve the confidence in the RfD. The low end of these values (i.e., 0.003 mg kg^−1^ day^−1^) is identical to the existing RfD in IRIS, suggesting that drinking water criteria based on an RfD of 0.003 mg kg^−1^ day^−1^ are sufficiently protective of human health. Importantly, the information gained from recent 2‐year bioassays and MOA research greatly improve the scientific basis for these toxicity criteria.

## Supporting information


**Table S1**. Portal Flux Dose Metrics
**Figure S1.** Tissue chromium levels in mice and rats exposed to Cr(VI) in drinking water for 90 days. Data (mean ± sd; *p < 0.05) taken from Kirman et al. (2012). Mm, mouse; Rn, liver
**Figure S2.** Comparison of concentrations employed in various reproductive/developmental toxicity studies with NTP (blue hatched boxes). Boxes represent range of concretions in a study; a solid line indicates only one dose was examined. The dotted line in each plot represents the LOAEL for diffuse epithelial hyperplasia in B6C3F1 mice in the NTP (2008) 2‐year bioassay. Note: Stanley et al. (2014) presented a plot indicating effects on follicles from 5 to 200 ppm Cr(VI); however, most of the detailed experiments were done with 50 ppm Cr(VI) (indicated by the dashed line in the box plot). De Flora, Iltcheva, and Balansky (2006) examined fetuses for genotoxicity (results were negative for exposure via drinking water).
**Table A.1.** Age‐, Sex‐, and Group‐Specific Dosing Parameters Calculated for Mice and Rats Under Conditions of the NTP Bioassay (NTP, 2008)
**Table A.2.** PBPK‐Derived Lifetime Average Daily Dose Estimates
**Figure A.1.** Depiction of Flux Values Used in the Cr(VI) Risk Assessment for SI Effects.
**Figure A.2** Example Calculation: Conversion of Mouse Sectional Flux to Mouse Pyloric Flux Using the PBPK Model
**Figure A.3** Example Calculation: Conversion of Sectional Flux to Human Equivalent Dose Using the PBPK Model
**Figure A.4.** Example Calculation: PBPK Modeling to Support DDEF Value
**Table S2.** Number of HTS Assay Endpoints Used to Evaluate Cr(VI) Bioactivity through the Tox21 Database, Organized according to Endocrine Disruption Categories^1^
