# Supplementary material for: Integration of mechanistic and pharmacokinetic information to derive oral reference dose and margin‐of‐exposure values for hexavalent chromium
Source: J Appl Toxicol. 2017 Oct 24;38(3):351–65. doi: 10.1002/jat.3545 (PMC5813206; doi:10.1002/jat.3545)
Supplement: Supplementary file 1 — Table S1. Portal Flux Dose Metrics Figure S1. Tissue chromium levels in mice and rats exposed to Cr(VI) in drinking water for 90 days. Data (mean ± sd; *p < 0.05) taken from Kirman et al. (2012). Mm, mouse; Rn, liver Figure S2. Comparison of concentrations employed in various reproductive/developmental toxicity studies with NTP (blue hatched boxes). Boxes represent range of concretions in a study; a solid line indicates only one dose was examined. The dotted line in each plot represents the LOAEL for diffuse epithelial hyperplasia in B6C3F1 mice in the NTP (2008) 2‐year bioassay. Note: Stanley et al. (2014) presented a plot indicating effects on follicles from 5 to 200 ppm Cr(VI); however, most of the detailed experiments were done with 50 ppm Cr(VI) (indicated by the dashed line in the box plot). De Flora, Iltcheva, and Balansky (2006) examined fetuses for genotoxicity (results were negative for exposure via drinking water). Table A.1. Age‐, Sex‐, and Group‐Specific Dosing Parameters Calculated for Mice and Rats Under Conditions of the NTP Bioassay (NTP, 2008) Table A.2. PBPK‐Derived Lifetime Average Daily Dose Estimates Figure A.1. Depiction of Flux Values Used in the Cr(VI) Risk Assessment for SI Effects. Figure A.2 Example Calculation: Conversion of Mouse Sectional Flux to Mouse Pyloric Flux Using the PBPK Model Figure A.3 Example Calculation: Conversion of Sectional Flux to Human Equivalent Dose Using the PBPK Model Figure A.4. Example Calculation: PBPK Modeling to Support DDEF Value Table S2. Number of HTS Assay Endpoints Used to Evaluate Cr(VI) Bioactivity through the Tox21 Database, Organized according to Endocrine Disruption Categories1 [file JAT-38-351-s001.pdf]

Supplemental Material

Table S1. Portal Flux Dose Metrics

Dose Group	Male Mice (portal flux)	Female Mice (portal flux)	Female Rat[b] (portal flux)
**Control[a]**	5.2-05	4.0E-05	4.7E-06
**1**	5.7E-02	4.3E-02	6.7E-03
**2**	1.2E-01	1.8E-01	2.5E-02
**3**	2.9E-01	4.0E-01	7.3E-02
**4**	5.0E-01	6.5E-01	1.4E-01

[a]Based on measured Cr(VI) in control tap water; doses 1–4 correspond to the Cr(VI) concentrations used in the NTP study.
[b]No male rat endpoints were modeled.

Figure S1.

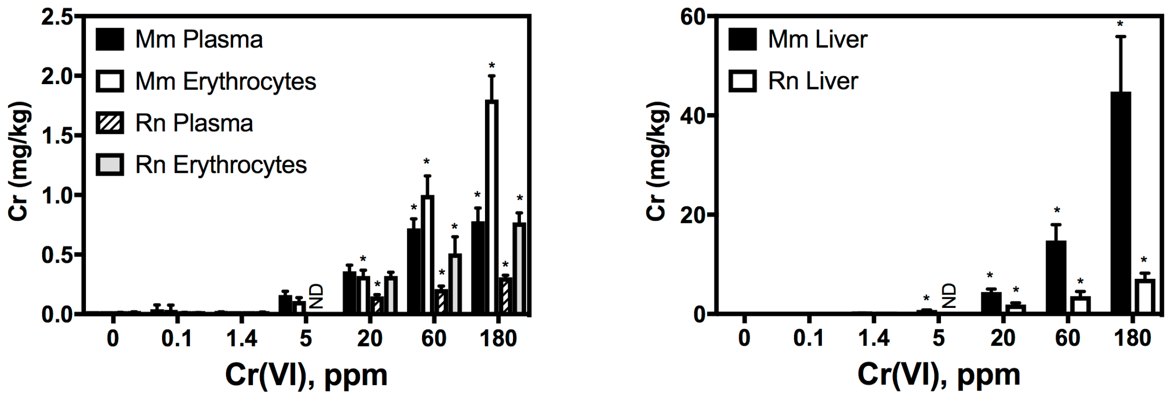

Figure S1. Tissue chromium levels in mice and rats exposed to Cr(VI) in drinking water for 90 days. Data (mean ± sd; *p<0.05) taken from Kirman et al. (2012). Mm, mouse; Rn, liver

Figure S2.

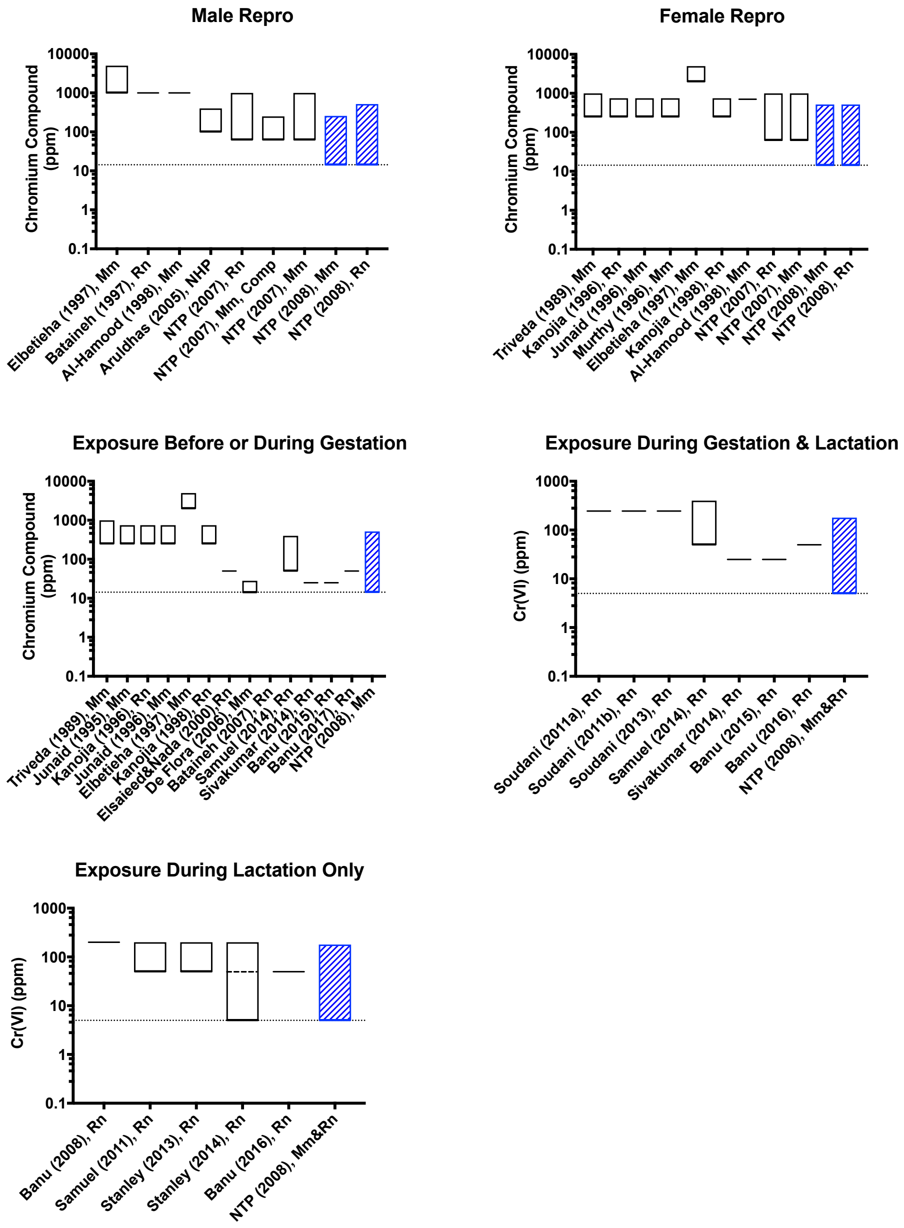

Figure S2. Comparison of concentrations employed in various reproductive/developmental toxicity studies with NTP (blue hatched boxes). Boxes represent range of concretions in a study; a solid line indicates only one dose was examined. The dotted line in each plot represents the LOAEL for diffuse epithelial hyperplasia in B6C3F1 mice in the NTP (2008) 2-year bioassay. Note: Stanley et al. (2014) presented a plot indicating effects on follicles from 5 to 200 ppm Cr(VI); however, most of the detailed experiments were done with 50 ppm Cr(VI) (indicated by the dashed line in the box plot). De Flora et al. (2006) examined fetuses for genotoxicity (results were negative for exposure via drinking water).

Appendix A. Calculation of Lifetime Average Daily Doses of Chromium Delivered to Mouse Small Intestines in the NTP Bioassay Using PBPK Modeling

A revised physiologically based pharmacokinetic (PBPK) model has been developed for chromium in rodents following oral exposures (Kirman et al., 2017). Although the toxicokinetic data collected as part of the cancer bioassay conducted by NTP (2008) are for systemic tissues and urinary excretion, which are not directly related to the target tissues of interest (i.e., small intestines, hepato-portal system tissues), because the target tissue also serves as the primary site of chromium absorption, the toxicokinetic data indicate the extent to which chromium has passed through the small intestines (i.e., Cr flux) and portal system (i.e., portal flux). All PBPK modeling was performed using the Microsoft Excel Add-In for acslX (AEgis TG).

A.1 PBPK Simulations for NTP Bioassay

Age-, sex- and group-specific values for chromium dose, drinking water consumption rate, and body weight were calculated from information provided in the bioassay (NTP, 2008) and are summarized in **Table A.1**. For calculating the lifetime average daily dose (LADD), the mouse and rat lifetimes were split into two time periods: (1) early life, for week 1-14; and (2) late life, for weeks 15-106. This was done to account for significant differences in the dose of Cr(VI) for the two time periods (early life>late life), based on drinking water rate and body weight information provided by NTP (2008). A time point of 14 weeks was selected since it reflects the mid-point between the toxicokinetic data collection time points for weeks 2 and 26 in the NTP bioassay. LADD values were calculated as time-weighted averages for five PBPK-derived measures of internal dose [i.e., estimates of Cr(VI) flux] (**Figure A.1**):

1. *Duodenum Flux (amtabsd6)* – Reflects the amount of Cr(VI) taken up into duodenum, per kg duodenum tissue per day.
2. *Jejunum Flux (amtabsj6)* – Reflects the amount of Cr(VI) taken up into jejunum, per kg jejunum tissue per day.
3. *Ileum Flux (amtabsi6)* – Reflects the amount of Cr(VI) taken up into ileum, per kg ileum jejunum per day.
4. *Pyloric Flux (amt2d6)* – Reflects the amount of Cr(VI) released from the stomach lumen to the small intestines lumen, per kg small intestines tissue per day.
5. *Portal Flux (amt2port6)* – Reflects the amount of Cr(VI) transported from the small intestines tissue and released to portal plasma, per kg body weight per day.

A depiction of all five dose measures is provided in **Figure A.1** and the predicted values are provided in **Table A.2**. The first three dose measures (SI sectional flux estimates) were used in the risk assessment to characterize the dose-response relationships (i.e., BMD modeling) for SI endpoints. Portal flux was used to characterize the dose-response relationship for systemic endpoints. Pyloric flux was used to support interspecies extrapolation from rodents to humans.

LADD values expressed in terms of SI section flux estimates are provided in **Table A.2** for each treatment group for male and female mice, and for female rats in the NTP bioassay. Estimates for SI sectional flux and portal flux were used to support benchmark dose (BMD) modeling.

A.2 PBPK Simulations for Interspecies Extrapolation

Points of departure, expressed in terms of SI sectional flux or portal flux, were converted to human equivalent doses using the rodent and human PBPK model as follows:

SI Sectional Flux => Total Sectional Flux => Pyloric Flux => Human Equivalent Dose

Portal Flux => Pyloric Flux => Human Equivalent Dose

Total Sectional Flux represents the sum of the three sectional flux values (duodenum+jejunum+ileum), so that total tissue risk is estimated. In this way, a point of departure (POD) based on a response rate of 5% would be allocated across the tissue (e.g., in the mouse: ~4.65% response in duodenum, 0.32% response in jejunum, and 0.028% response in ileum based upon their relative contribution to total sectional flux). This approach assumes that for a given value of pyloric flux, the dose of Cr(VI) delivered to the small intestines and to systemic tissues, as well as their associated risks, are equivalent for all species. An example conversion of the POD values expressed in terms of SI section flux to pyloric flux is provided below:

1. The POD value (BMDL05) of 1.1 mg/kg-day (in terms of SI sectional flux) was divided by an EF_{AD} factor of 3 to yield a value of 0.37 mg/kg SI-day (in terms of SI sectional flux).
2. The mouse PBPK model was used to identify the value for pyloric flux when the value for total sectional flux is equal to 0.37 mg/kg SI-day (**Figure A.2)**. This occurs when pyloric flux is also equivalent to a value of 0.37 mg/kg SI-day.
3. Pyloric flux estimates were converted to human equivalent dose (oral administered in terms mg/kg BW-day) using the human PBPK model. The human PBPK model predicts that pyloric flux will be equal to 0.37 mg/kg-day, when the long-term oral dose is equal to 0.02 mg/kg BW-day (**Figure A.3**). Value for human equivalent doses for this extrapolation reflect the lifetime average daily dose (LADD) across age groups, as defined in the main body of the paper.

A.3 PBPK Simulations to Support Data-Derived Extrapolation Factor

A data-derived extrapolation factor to account for human toxicokinetic variation was calculated using the ratio of internal doses predicted by the PBPK model for a potential sensitive subgroup (hypochlorhydria) to the average adult:

DDEF = Pyloric flux (hypochlorhydria) / Pyloric flux (average adult)

Because reducing agents in the gastrointestinal tract are depleted by Cr(VI) in a dose-dependent manner, the DDEF value derived using the PBPK will depend upon the value of the POD for each endpoint. An example calculation is provided in **Figure A.4**.

A.4 References

Kirman, C. R., Hays, S. M., Aylward, L. L., Suh, M., Harris, M. A., Thompson, C. M., Haws, L. C. and Proctor, D. M. (2017). Physiologically based pharmacokinetic model for rats and mice orally exposed to chromium. Toxicol Sci. Submitted

NTP (2008). National Toxicology Program technical report on the toxicology and carcinogenesis studies of sodium dichromate dihydrate (CAS No. 7789-12-0) in F344/N rats and B6C3F1 mice (drinking water studies), NTP TR 546. NIH Publication No. 08-5887.

Table A.1. Age-, Sex-, and Group-Specific Dosing Parameters Calculated for Mice and Rats Under Conditions of the NTP Bioassay (NTP, 2008)

		Parameter Values for Calculating LADD					
		Weeks 1-14			Weeks 15-106		
Sex, Species	Treatment Group (mg SDD/L)	Dose (mg Cr(VI)/kg-day)	Drinking Water Rate (L/hr/kg)	Body Weight (kg)	Dose (mg Cr(VI)/kg-day)	Drinking Water Rate (L/hr/kg)	Body Weight (kg)
Female Mouse	14.3	0.67	0.0054	0.024	0.24	0.0020	0.057
	57.3	2.6	0.0053	0.024	0.94	0.0019	0.055
	172	6.5	0.0044	0.023	2.7	0.0018	0.051
	516	14.8	0.0034	0.022	7.9	0.0018	0.045
Male Mouse	14.3	0.58	0.0046	0.033	0.43	0.0036	0.052
	28.6	1.2	0.0046	0.033	0.88	0.0037	0.052
	85.7	3.3	0.0045	0.032	2.3	0.0032	0.052
	257.4	7.9	0.0036	0.029	5.4	0.0025	0.049
Female Rat	14.3	0.42	0.0040	0.16	0.29	0.0026	0.24
	57.3	1.62	0.0039	0.16	1.13	0.0025	0.24
	172	4.13	0.0033	0.16	3.00	0.0023	0.23
	516	11.28	0.0030	0.16	8.22	0.0021	0.23

Table A.2. PBPK-Derived Lifetime Average Daily Dose Estimates

Sex, Species	Weeks	Treatment Group (mg SDD/L)	Duodenal Flux (amtabsd6)	Jejunal Flux (amtabsj6)	Ileum Flux (amtabsi6)	Portal Flux (amt2port6)	Pyloric Flux (amt2d6)
Female mouse	1-14	14.3	15.0	1.5	0.15	0.21	8.1
		57.3	46.7	10.9	1.6	0.80	42.4
		172	73.1	19.6	3.1	1.3	129
		516	89.6	22.8	3.6	1.6	324
	15-106	14.3	1.2	0.10	0.0093	0.017	1.9
		57.3	5.4	0.71	0.076	0.088	10.2
		172	15.0	3.2	0.45	0.26	41.5
		516	27.2	6.9	1.1	0.51	152
	1-106	14.3	3.0	0.29	0.028	0.043	2.7
		57.3	10.9	2.1	0.28	0.18	14.4
		172	22.7	5.4	0.81	0.40	53.1
		516	35.4	9.0	1.4	0.65	175
Male mouse	1-14	14.3	10.9	1.2	0.11	0.16	6.6
		28.6	20.7	3.4	0.40	0.32	15.6
		85.7	43.1	10.7	1.6	0.76	56.5
		257.4	61.3	16.3	2.6	1.1	156
	15-106	14.3	2.8	0.29	0.028	0.042	4.6
		28.6	5.6	0.72	0.076	0.089	10.2
		85.7	13.3	2.7	0.37	0.22	34.3
		257.4	22.2	5.5	0.86	0.40	98.6
	1-106	14.3	3.9	0.41	0.039	0.057	4.9
		28.6	7.6	1.1	0.12	0.11	10.9
		85.7	17.2	3.7	0.54	0.29	37.3
		257.4	27.4	7.0	1.1	0.48	106
Female rat	1-14	14.3	0.87	0.0054	0.00012	0.0018	2.9
		57.3	11.0	0.33	0.024	0.025	24.8
		172	24.4	2.7	0.35	0.080	98.0
		516	32.1	7.5	1.3	0.15	366
	15-106	14.3	1.7	0.012	0.00026	0.0073	1.6
		57.3	8.8	0.38	0.018	0.024	12.9
		172	12.8	2.5	0.37	0.070	61.0
		516	14.0	3.8	0.68	0.13	245
	1-106	14.3	1.6	0.012	0.00024	0.0065	1.8
		57.3	9.1	0.37	0.018	0.024	14.4
		172	14.3	2.6	0.36	0.072	65.9
		516	16.4	4.3	0.77	0.14	261

*All internal flux estimates are expressed in terms of mg Cr(VI)/kg tissue (SI section or total) per day.

Fig. A.1. Depiction of Flux Values Used In the Cr(VI) Risk Assessment for SI Effects

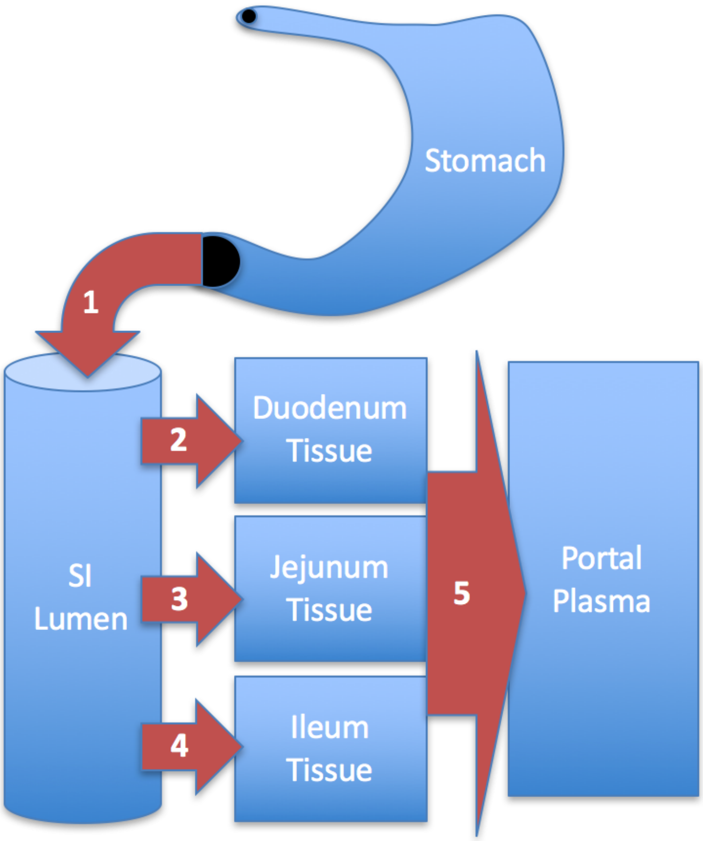

1 = pyloric flux (mg Cr(VI)/kg total SI-day) used for interspecies extrapolation;
2-4 = SI segment flux (mg Cr(VI)/kg SI segment-day) used for dose-response modeling of mouse data;
5 = Portal flux (mg Cr(V))/kg BW-day) used for interspecies extrapolation; calculated as the segment mass-weighted average of flux #1

Fig. A.2 Example Calculation: Conversion of Mouse Sectional Flux to Mouse Pyloric Flux Using the PBPK Model

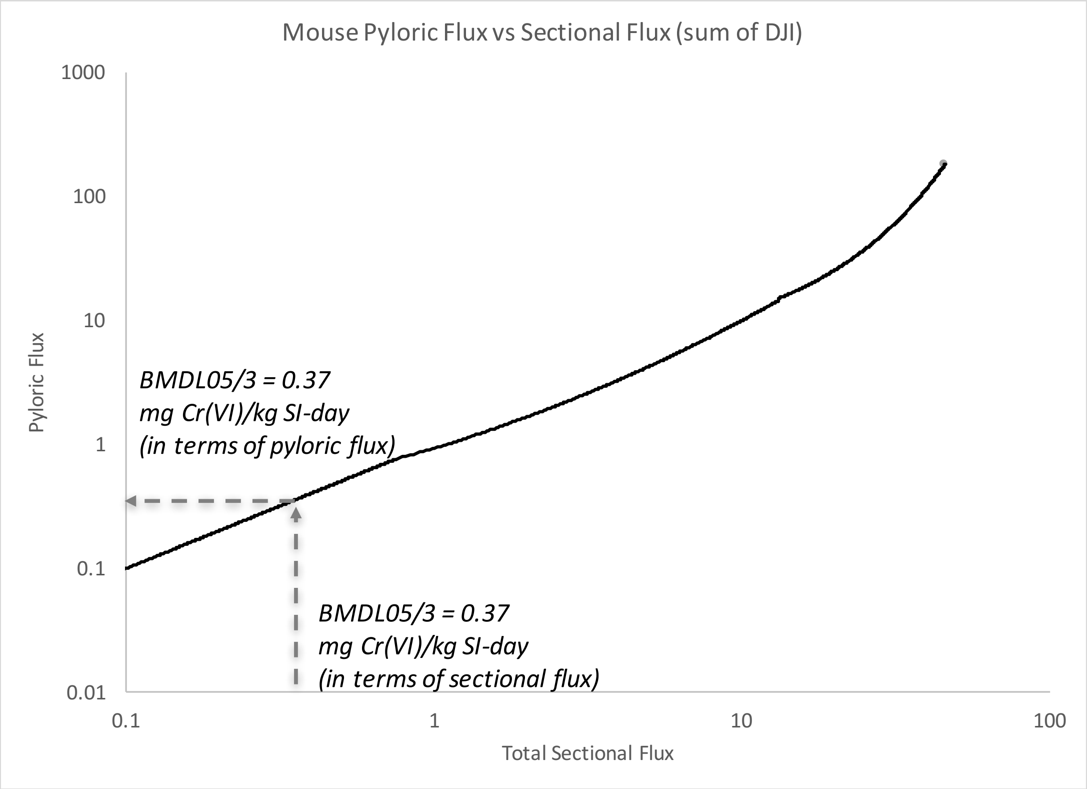

Fig. A.3 Example Calculation: Conversion of Sectional Flux to Human Equivalent Dose Using the PBPK Model

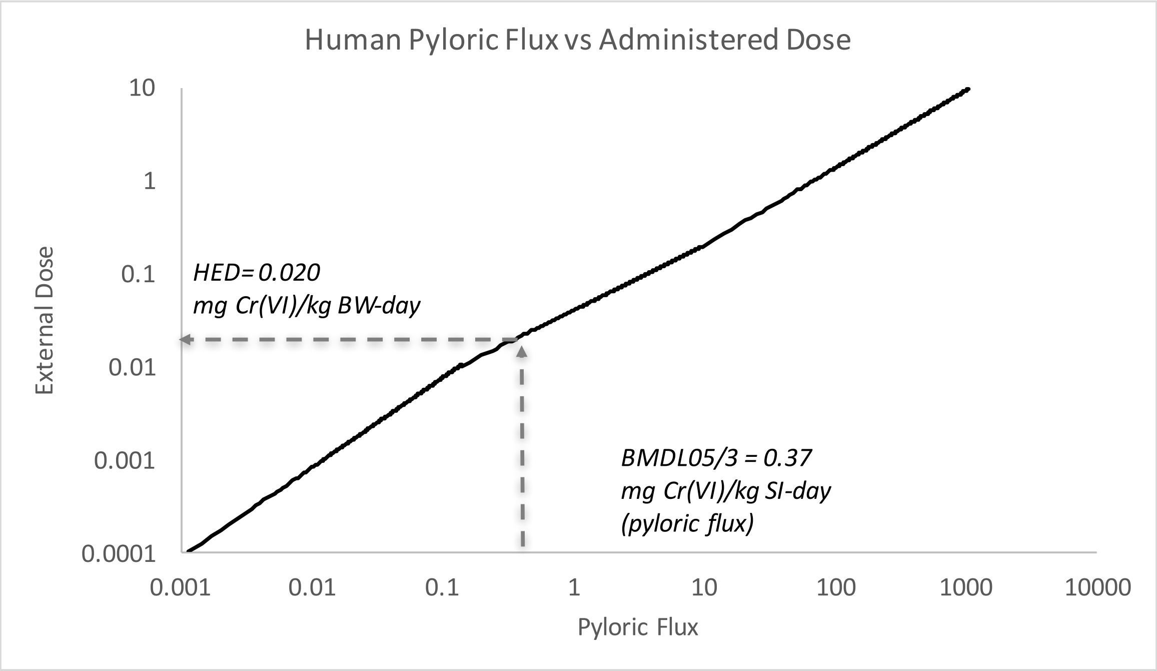

Fig. A.4. Example Calculation: PBPK Modeling to Support DDEF Value

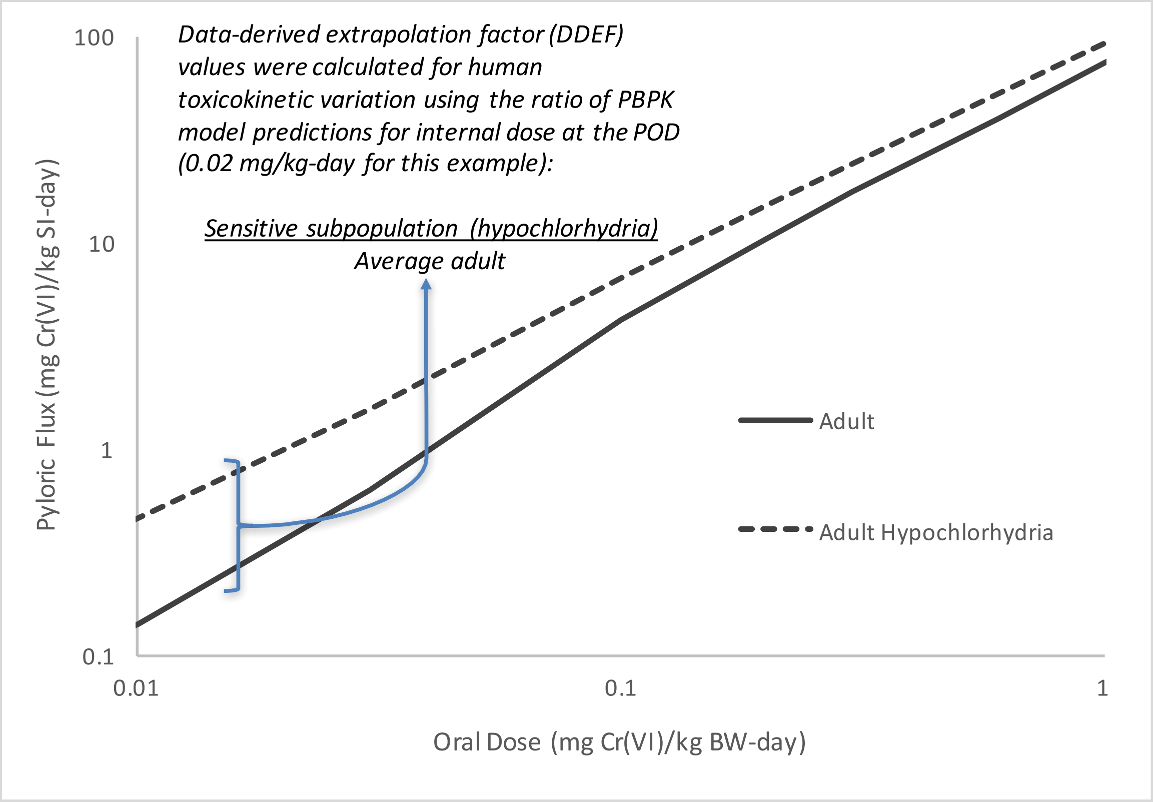

Appendix B: Evaluation of Cr(VI) as an Endocrine Disrupter Using Tox21 Consortium Data

Methods

In vitro high-throughput screening (HTS) data are available for Cr(VI) (as sodium dichromate dihydrate [SDD]) through the Tox21 consortium, a federal collaboration between the National Toxicology Program at the National Institute of Environmental Health Sciences and the National Center for Advancing Translational Sciences, the Food and Drug Administration, and the United States Environmental Protection Agency (U.S. EPA). To place HTS findings in the context of potential endocrine disruption, data were first queried through the Endocrine Disruption Screening Program for the 21st Century (EDSP21) Dashboard (U.S. EPA 2016). The EDSP21 Dashboard includes data that incorporate overall weight of evidence for determining androgen receptor (AR), estrogen receptor (ER), and thyroid receptor (TR) activity, as represented by an area under the curve (AUC) value, which integrates assay endpoint activity across all AR, ER, or TR relevant assays (defined by Judson et al., 2015). However, this dashboard is not updated as frequently as other websites containing Tox21 data, and no assay information was available for SDD through the EDSP21 Dashboard at the time of the analysis.

Tox21 HTS data, however, were available through the U.S. EPA's ToxCast and Tox21 Summary Files (invitrodb_v2, released Oct 2015) (U.S. EPA, 2015). Data from this website included summary-level assay statistics, including summary activity ("hit") calls, represented by values of 1 (active), 0 (inactive), or –1 (activity could not be determined), as defined previously (Judson et al., 2015, 2016). AC_{50} values were also included, indicating the concentration at which the activity reached 50% of the its maximal values for an assay-chemical pair. Z-scores were also used to evaluate the relationship between chemical-assay activity and assay cytotoxicity distributions, where a higher Z-score represented assay activity that occurred at concentrations far below the cytotoxicity threshold (Judson et al., 2016). Active chemical-assay pairs were required to have Z-scores >2 to account for potential cytotoxic signal burst interference, similar to a recent ToxCast data evaluation (Auerbach et al., 2016). Results were organized by endocrine disruption target categories, specifically AR, ER, and TR, as defined through EDSP21 (U.S. EPA, 2016).

Results

HTS Tox21 data were available on SDD across 113 assay endpoints. A subset of these mapped to the endocrine disruption target categories AR (4 assays), ER (4 assays), and TR (2 assays). Of all of the evaluated assay endpoints, 40 showed activity resulting from SDD treatment based on hit calls, two of which mapped to AR, one to ER, and one to TR. However, after considering the potential influence of cytotoxicity interference through a Z-score filter, 20 active assay endpoints remained, including only one assay endpoint mapping to the endocrine disruption target categories (**Table S2**). This assay endpoint was titled TOX21_TR_LUC_GH3_Antagonist and mapped to TR. This assay used a rat pituitary gland cell line (GH3) dosed with varying concentrations (0 to 100 μM) of SDD over 28 hours. Luciferase induction was measured to detect transcriptional gene expression changes due to antagonist activity regulated by the human thyroid hormone receptor, alpha (THRA), and thyroid receptor, beta (THRB). The resulting AC_{50} and Z-score for this chemical-assay pair were 0.85 μM and 7.64, respectively.

Table S2. Number of HTS Assay Endpoints Used to Evaluate Cr(VI) Bioactivity through the Tox21 Database, Organized according to Endocrine Disruption Categories[1]

Endocrine Disruption Category Name	Number of Assays[2] Evaluated	Number of Assays that Showed SDD-Induced Activity	Number of Assays that Showed SDD-Induced Activity and Z-score >2
Androgen Receptor	4	2	0
Estrogen Receptor	4	1	0
Thyroid Receptor	2	1	1
Not Mapped to Endocrine Disruption Category	103	36	19
**Grand Total**	**113**	**40**	**20**

[1] Endocrine disruption categories were identified through the EDSP21 Dashboard (U.S. EPA 2016).
[2] Assays refer to assay endpoints.

References

Auerbach S, Filer D, Reif D, Walker V, Holloway AC, Schlezinger J, Srinivasan S, Svoboda D, Judson R, Bucher JR, Thayer KA. Prioritizing Environmental Chemicals for Obesity and Diabetes Outcomes Research: A Screening Approach Using ToxCast™ High-Throughput Data. Environ Health Perspect. 2016 Aug;124(8):1141-54.

Judson R, Houck K, Martin M, Richard AM, Knudsen TB, Shah I, Little S, Wambaugh J, Setzer RW, Kothiya P, Phuong J, Filer D, Smith D, Reif D, Rotroff D, Kleinstreuer N, Sipes N, Xia M, Huang R, Crofton K, Thomas RS. Analysis of the Effects of Cell Stress and Cytotoxicity on In Vitro Assay Activity Across a Diverse Chemical and Assay Space. Toxicol Sci. 2016 Oct;153(2):409.

Judson RS, Magpantay FM, Chickarmane V, Haskell C, Tania N, Taylor J, Xia M, Huang R, Rotroff DM, Filer DL, Houck KA, Martin MT, Sipes N, Richard AM, Mansouri K, Setzer RW, Knudsen TB, Crofton KM, Thomas RS. Integrated Model of Chemical Perturbations of a Biological Pathway Using 18 In Vitro High-Throughput Screening Assays for the Estrogen Receptor. Toxicol Sci. 2015 Nov;148(1):137-54.

U.S. EPA (2015). ToxCast & Tox21 Summary Files from invitrodb_v2. Available at: https://www.epa.gov/chemical-research/toxicity-forecaster-toxcasttm-data. [Accessed 4 Nov 2015].

U.S. EPA (2016). EDSP21 Dashboard. Available at: https://actor.epa.gov/edsp21. [Accessed on 3 Nov 2016].
